# Methods for Dealing With Missing Covariate Data in Epigenome-Wide Association Studies

**DOI:** 10.1093/aje/kwz186

**Published:** 2019-09-05

**Authors:** Harriet L Mills, Jon Heron, Caroline Relton, Matt Suderman, Kate Tilling

**Keywords:** Accessible Resource for Integrated Epigenomics Studies, Avon Longitudinal Study of Parents and Children, epigenetic data, imputation, missing data

## Abstract

Multiple imputation (MI) is a well-established method for dealing with missing data. MI is computationally intensive when imputing missing covariates with high-dimensional outcome data (e.g., DNA methylation data in epigenome-wide association studies (EWAS)), because every outcome variable must be included in the imputation model to avoid biasing associations towards the null. Instead, EWAS analyses are reduced to only complete cases, limiting statistical power and potentially causing bias. We used simulations to compare 5 MI methods for high-dimensional data under 2 missingness mechanisms. All imputation methods had increased power over complete-case (C-C) analyses. Imputing missing values separately for each variable was computationally inefficient, but dividing sites at random into evenly sized bins improved efficiency and gave low bias. Methods imputing solely using subsets of sites identified by the C-C analysis suffered from bias towards the null. However, if these subsets were added into random bins of sites, this bias was reduced. The optimal methods were applied to an EWAS with missingness in covariates. All methods identified additional sites over the C-C analysis, and many of these sites had been replicated in other studies. These methods are also applicable to other high-dimensional data sets, including the rapidly expanding area of “-omics” studies.

In medical research, we are increasingly dealing with high-dimensional data sets detailing exposures, covariates, and outcomes. This creates challenges for scaling up standard statistical methods—in terms of plausibility of underlying assumptions, but also in terms of practicalities such as computing time. An example of this is the use of multiple imputation (MI) for dealing with missing data, where an approach that is practicable for a data set with a small number of variables (fitting an imputation model 100 times for each variable and combining the results) may not be practicable for a high-dimensional data set. Many methods for MI have been explored (1–4), but few were designed to handle data sets with over 500 covariates or in which the number of covariates is much larger than the number of cases (5–7). Additionally, though MI packages (e.g., *mice* in R (R Foundation for Statistical Computing, Vienna, Austria) (8) and *ice* in Stata (StataCorp LLC, College Station, Texas) (9)) exist, these packages were not designed for efficiency with high-dimensional data sets. Therefore, there is a need for new, efficient methods with which to implement MI in high-dimensional data sets with missingness.

A commonly encountered high-dimensional problem is that of epigenetic studies evaluating DNA methylation—a reversible chemical modification of DNA whereby a methyl group is added to a cytosine nucleotide. These studies measure methylation at 480,000 or 850,000 CpG sites (per person studied), and a few recent studies have evaluated millions of CpG sites, though with small sample sizes (10, 11). To investigate hypotheses about the association of DNA methylation with a specific phenotype, DNA methylation measurements across the genome are tested for associations with the phenotype (often called an epigenome-wide association study (EWAS)) by repeatedly fitting a simple univariate model for each CpG site—thus fitting 480,000 models in total.

Missingness in EWAS can occur in the methylation measures or the covariates. However, missingness in the methylation measures tends to be minimal and to relate to technical issues of data generation. Missingness in the covariates, by contrast, tends to have a much greater impact on the analysis, causing decreased statistical power. Here we consider only missingness in the covariates. Commonly, a complete-case (C-C) analysis is used for an EWAS, where only cases with complete data on the outcome and all covariates are analyzed. This will reduce the power of the analysis (in one example, the number of cases included was reduced from 1,018 to 678 (12)) and be biased if the chance of being a complete case is associated with the outcome, given the covariates in the model (2, 13–17). For example, if smokers were less likely to attend a clinic at which samples were taken for epigenetic analysis, and so were people with higher body mass index, this would induce collider bias between smoking and body mass index in a C-C analysis (18). This would mean that in a C-C EWAS, smoking would tend to be associated with all methylation sites that were affected by body mass index and vice versa.

MI can be used to minimize bias and inefficiency in the presence of incomplete data. MI works by specifying prediction models for each variable with missingness (3). In order to avoid bias towards the null, these models need to include all variables in the analysis model (see simulation in Web Appendix 1, Web Tables 1 and 2, and Web Figures 1 and 2, available at https://academic.oup.com/aje), plus any auxiliary variables (16, 19). For an EWAS, this would mean imputing the missing covariate data using all CpG sites, which would be computationally intensive, and would require additional methods if the number of CpG sites (typically ≥480,000) was greater than the number of cases (typically, at most, a few thousand).

Here we used simulations and an applied example to explore different methods for imputing missing values of covariates. Initially we used the computationally intensive method of imputing using each CpG site in turn. We extended this method by using groups of CpG sites together to impute data on the missing variable, reducing the computational time. These groups were entirely randomly selected or were designed to include some systematically selected CpG sites—in this instance, using CpGs determined to be associated with the missing variable (building on work by Wu et al. (20)). We compared the imputation methods with regard to their standard error, bias, and computation time. Our conclusions are also useful for researchers analyzing other high-dimensional data sets, including the rapidly expanding area of “-omics” studies.

## METHODS

### Simulation study

We used a publicly available data set describing DNA methylation at 482,739 CpG sites obtained using the Infinium human methylation 450k array (Infinium HumanMethylation450 BeadChip kit; Illumina, Inc., San Diego, California) (21) for 464 persons (22), downloaded from the National Center for Biotechnology Information’s Gene Expression Omnibus (23). Data on age, sex, and smoking status (never, former, or current smoker) were provided for every individual. Methylation measures were standardized for each CpG.

Covariates in this data set had no missing data; therefore, missingness for smoking status was induced using 2 missingness mechanisms (MMs): 1) MM1—missing with probability 75% for males aged 57 years or over; and 2) MM2—missing with probability 50% for males aged 57 years or over and with probability 12.5% for all remaining persons. These were both missing-at-random scenarios, where missingness depended on the completely observed covariates age and sex, so that both a C-C analysis and MI including these variables as covariates would be unbiased. The percentages used ensured a comparable proportion of missingness for both mechanisms.

### Imputation methods

All imputation models included the covariates age and sex; smoking was imputed using the “polyreg” method in *mice*, which uses polytomous logistic regression. We generated 100 sets of imputed data for each imputation. Use of 100 imputations is very conservative—recent literature has suggested that the number of imputations should be equivalent to the percentage of missing data (a linear rule (24, 25)) or that the number is better approximated by a quadratic rule (26). Five imputation methods were used (described below and in Table 1).

**Table 1.** Characteristics and Performance of Different Imputation Methods Used to Impute Smoking Status From Epigenetic Data^a^

Imputation Method	Characteristics of Method		Characteristics of Results			
	No. of Imputation Procedures	Imputation Model	True Positives	False Positives	Bias	Speed (1 = Fastest)
Complete-case	0	N/A	Poor	Very good	Unbiased^b^	1
Separate CpG sites	482,739	Smoking ~ single CpG site + age + sex	Good	Good	Unbiased	8
Random bins (3:1)	3,219^c^	For each bin: smoking ~ 150 CpG sites + age + sex	Good	Poor	Unbiased	6
Random bins (10:1)	10,728^c^	For each bin: smoking ~ 45 CpG sites + age + sex	Good	Good	Unbiased	4
Naive method	1	Smoking ~ C-C CpG sites + age + sex	Good	Poor	Biased towards the null for non–C-C CpG sites	3
Wu method	1	Smoking ~ selected CpG sites + age + sex	Good	Good	Biased towards the null for nonselected CpG sites	2
Wu bins (3:1)	3,353^c,d^	For each bin: smoking ~ 150 CpG sites (including Wu-selected CpG sites) + age + sex	Good	Poor	Unbiased	7
Wu bins (10:1)	12,378^c,d^	For each bin: smoking ~ 45 CpG sites (including Wu-selected CpG sites) + age + sex	Good	Good	Unbiased	5

Abbreviations: C-C, complete-case; N/A, not applicable.

^a^ This table provides details on the imputation methods described in the text and their results for the simulations only. *N*_CpG_ is the number of CpG sites included in the analysis (*N*_CpG_ = 482,739).

^b^ Methods classified as “unbiased” in this table are only unbiased if the imputation model is correct and data are missing at random.

^c^ This is approximately *N*_CpG_/bin size.

^d^ Recall that the bins for the “Wu bins” method always contain the subset of CpG sites selected in the forward-stepwise selection process, so there are slightly more bins for the “Wu bins” method than for the “random bins” method, in order to accommodate the extra sites.

#### Separate CpG sites

For each CpG site in turn, smoking status was imputed using age, sex, and methylation measure at that site. The 100 imputed data sets for each site were pooled for the EWAS for each site, using standard MI methods to obtain standard errors for the coefficients (27).

#### Random bins of fixed size

CpG sites were divided into bins of fixed size. Smoking status was imputed for each bin using age, sex, and methylation measure at all of the sites in the bin. The 100 imputed data sets per bin were pooled for the EWAS analyses for CpG sites in that bin (27). Two bin sizes were used—150 and 45—reflecting approximate 3:1 and 10:1 ratios of cases (i.e., individuals) to variables, respectively (28, 29), and resulting in 3,219 and 10,728 bins, respectively (Table 1).

The random-bins method is a compromise between using every CpG in a single bin for the imputation and imputing using single CpGs in turn (the first method described above). Both are computationally intensive, with the former also having more variables than cases, meaning that computations using the imputation model would not run without additional methods. Randomly assigning the CpGs into bins maximizes the information being used for each imputation while also improving the calculation time. Other studies have also used bins to overcome the problem of many covariates (that of Yin et al. (30) being one example).

#### Using associated CpG sites—naive method

One MI procedure was carried out, imputing missing data on smoking status from age, sex, and methylation measures for the set of CpG sites that were significantly associated with smoking in the C-C analysis. The 100 imputed data sets were pooled for the EWAS for all CpG sites (27).

#### Using associated CpG sites—Wu method

A forward-stepwise selection model was used to select a final set of CpG sites to be included in the imputation model, from the top 100 associated CpG sites identified from the C-C analysis (using the Bayesian Information Criterion, as in Wu et al. (20)). One MI procedure was carried out, imputing missing data on smoking status from age, sex, and methylation measures at all of the selected CpG sites. The 100 imputed data sets were pooled for the EWAS for all CpG sites (27).

#### Random bins of fixed size always including associated CpG sites—Wu bins

The random binning and Wu methods were combined such that the set of CpG sites selected by the Wu method was included in every bin alongside randomly selected sites. Smoking status was imputed for each bin using age, sex, and methylation measure at all sites in the bin. The 100 imputed data sets for the bin were pooled for the EWAS for CpG sites in that bin (27). As before, 2 bin sizes were used—150 and 45 (including the selected sites and the random sites), resulting in 3,353 and 12,378 bins, respectively (Table 1).

Ten data sets with missingness were generated for each of the 2 MMs and were used to perform 10 repeats of each imputation method for each mechanism. Only 10 repeats were performed because imputation and regression in such a high-dimensional data set were slow and computationally intensive. With only 10 repeats, conclusions may be distorted by sampling variability. To confirm conclusions, we reduced the data set to 2,000 CpG sites (removing 480,739 sites) (see Web Appendix 2) and performed 1,000 repeats.

All imputation and analyses were performed on the University of Bristol’s high-performance computer. Imputation and result pooling were performed with the *mice* and *survey* packages in R (31), using Rubin’s rules (15, 27).

The “complete data set” is the data set without missingness, and the EWAS on these data gave the “truth”: 298 CpG sites associated with (current and former) smoking. The best-performing method should have a high true-positive rate (the percentage of “true” sites correctly identified as significant by the method) and a low false-positive rate (the percentage of sites identified as significant by the method which were not “true” sites). Low computing time would also be advantageous. These performance measures were reported for each method, alongside the bias in the coefficients as compared with the “truth.”

### Epigenome-wide association studies

A linear regression analysis was used, relating age, sex, and smoking status to the methylation measure at each CpG site (an EWAS):

CpG ~ age + sex + smoking.

Note that this model is deliberately simplistic and does not adjust for any other covariates (such as batch effects or other confounders relevant to smoking), since the imputation methods are the focus of this simulation study.

A Bonferroni correction was used to identify those CpG sites associated with (current or former) smoking with *P* < 0.05/*N*_CpG_, where *N*_CpG_ is the number of CpG sites.

The EWAS was performed on the complete data set (i.e., 464 cases with information on smoking, age, and sex and DNA methylation measures at 482,739 sites) to obtain a set of results representing “the truth”: the 298 CpG sites associated with smoking when there was no missingness. Additionally, C-C EWAS were performed for each data set with missingness (i.e., using only those cases with complete data).

### Application to an EWAS of smoking in pregnancy

We applied the imputation methods to data from the Avon Longitudinal Study of Parents and Children (ALSPAC) to illustrate their use with real missing data across multiple covariates. ALSPAC initially recruited 14,541 pregnant women resident in Avon, United Kingdom, with expected delivery dates of April 1, 1991–December 31, 1992 (32, 33); follow-up increased this to 15,247 pregnancies. Detailed follow-up of the mothers and children has provided a rich set of self-reported data, linked medical records, and data collected at health clinics. The study website contains details on all of the data that are available through a fully searchable data dictionary (34). Ethical approval for the study was obtained from the ALSPAC Ethics and Law Committee and local research ethics committees. In a substudy, Accessible Resource for Integrated Epigenomics Studies (ARIES), investigators selected approximately 1,000 mother-child pairs and profiled DNA methylation from samples collected at multiple time points in both mother and child (35).

DNA methylation was measured from blood collected at the ALSPAC Focus on Mothers clinic (974 cases). An EWAS explored the relationship between maternal smoking status and DNA methylation, including the following confounders believed to be associated with smoking and DNA methylation: age of the mother (at birth of the child), parity, maternal educational level, housing tenure, and social class; batch effects were also included. Data collected around the time of the child’s birth have fewer than 5% missing values for persons in ARIES; however, here information on maternal smoking status was obtained from a later questionnaire (around 18 years after the birth of the ALSPAC child), intentionally producing high missingness (34.6%) (Web Table 3).

Three methods (random bins, Wu, and Wu bins) were applied to the ARIES data set to impute missing data for all variables. A bin size of 95 was used (10:1 ratio of cases to variables). These methods were chosen because they performed well in the simulations. Offspring birth weight, maternal alcohol intake during pregnancy, and maternal smoking reported at 18 weeks of pregnancy were also used in the imputation model but not in the EWAS. The EWAS results following imputation were compared with a C-C analysis and with a review of other smoking EWAS (36).

## RESULTS

### Simulation study

The individuals in the simulation data set were 70.5% male, with a mean age of 55.4 years (median, 56 years); 38.6% were nonsmokers, 56.7% were former smokers, and the remaining 4.7% were current smokers (Web Appendix 3, Web Table 4) (22). The MMs (MM1 and MM2; see Methods section) produced approximately 22% missingness for smoking status (Web Table 4). As intended, missingness varied by age and sex (see example in Web Figure 3).

**Figure 1.**
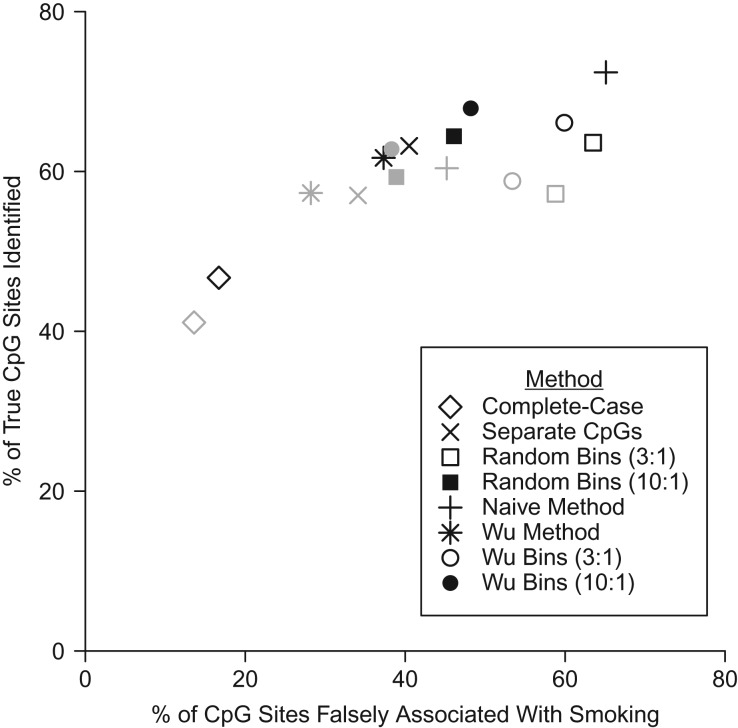
True-positive and false-positive percentages of CpG sites identified by different imputation methods in 10 repeats on the full (unreduced) data set. Values are listed in Table 2. Black symbols are for missingness mechanism 1, and gray symbols are for missingness mechanism 2.

For every imputation method, we report the true-positive (percentage of “true” sites correctly identified as significant by the method) and false-positive (percentage of sites identified as significant by the method which were not “true” sites) rates, the mean standard errors across β coefficients for the CpG sites (for former smokers), and computational time (Table 2, Figure 1, Web Table 5). Because of the way we designed the study, the C-C, separate CpGs, random bins, and Wu bins methods should be unbiased (as compared with the EWAS on the data set with no missingness) (Web Tables 6 and 7, Web Figures 4 and 5). The mean standard error for the separate CpGs method was not that much smaller than that for the C-C method, indicating minimal improvement. However, the mean standard error decreased as more information was added to the imputation model—see, for example, the decrease in standard error from small bins (10:1) to large bins (3:1).

**Table 2.** Performance of Different Imputation Methods for Imputing Smoking Status, Assessed by Comparing the EWAS on the Resulting Data Sets With an EWAS on the Complete Data^a^

Imputation Method	MM1						MM2						Time Relative to Separate CpG Method
	Associated CpG Sites					Mean SE of β Coefficients (SD)	Associated CpG Sites					Mean SE of β Coefficients (SD)	
	Total No. of Sites	No. in Complete Data Set	% of Complete Data Set	No. Not in Complete Data Set	% of Total Found		Total No. of Sites	No. in Complete Data Set	% of Complete Data Set	No. Not in Complete Data Set	% of Total Found		
Complete data^b^	298.0					0.0953 (0.0081)	298.0					0.0953 (0.0081)	
Complete-case	169.7	139.3	46.7	30.4	16.7	0.1073 (0.0099)	147.1	122.5	41.1	24.6	13.6	0.1069 (0.0099)	0.002
Separate CpG sites	330.0	188.3	63.2	141.7	40.5	0.1052 (0.0093)	282.8	169.9	57.0	112.9	34.1	0.1049 (0.0093)	1.000
Random bins (3:1)	537.2	189.6	63.6	347.6	63.5	0.0997 (0.0086)	482.2	170.5	57.2	311.7	58.8	0.0985 (0.0085)	0.517
Random bins (10:1)	373.6	192.0	64.4	181.6	46.1	0.1031 (0.0090)	326.3	176.6	59.3	149.7	38.9	0.1028 (0.0090)	0.339
Naive method	863.3	215.8	72.4	647.5	65.1	0.0984 (0.0084)	433.9	180.1	60.4	253.8	45.2	0.0974 (0.0083)	0.069
Wu method	312.0	183.8	61.7	128.2	37.3	0.1002 (0.0087)	290.4	170.8	57.3	119.6	28.2	0.1001 (0.0087)	0.059
Wu bins (3:1)	516.7	196.9	66.1	319.8	59.9	0.0984 (0.0084)	432.6	175.2	58.8	257.4	53.4	0.0972 (0.0083)	0.527
Wu bins (10:1)	410.2	202.2	67.9	208.0	48.2	0.0996 (0.0086)	349.2	187.1	62.8	162.1	38.3	0.0995 (0.0086)	0.412

Abbreviations: EWAS, epigenome-wide association study; MM, missingness mechanism; SD, standard deviation; SE, standard error.

^a^ The table shows the number of CpG sites associated with former or current smoking that were identified as significant in the regression analysis for each method, for both MMs. We report the number of these sites which were also significant in the EWAS on the complete data set (presented with the percentage, i.e., the true-positive rate) and the number of those which were not significant in the EWAS on the complete data set (presented with the percentage of those found to be significant which were “incorrect,” i.e., the false-positive rate). The mean and SD of the SEs (for the coefficients for the association of each CpG site with being a former or current smoker) are reported for each method. Note that this is the mean value across repeats of the mean and SD of the SEs within each repeat. Recall that the analysis on the complete data and C-C analysis did not require any imputation, making their computation time very low. Relative times are calculated from computation times averaged over example runs for MM1 and MM2; raw times are provided in Web Table 5. The table shows the results of the 10 repeats on the full (unreduced) data set.

^b^ Reference model.

The CC analysis had low statistical power for both missingness mechanisms (MM1 and MM2). Results for MM2 were similar to those of MM1, though fewer associated CpG sites were found for most methods, giving correspondingly lower true-positive and lower false-positive rates than for MM1 (Table 2). The separate CpGs method was computationally intensive, and it correctly identified only 63.2% of the sites that were associated with smoking in the complete data set (the true positives), with 40.5% of sites being false positives for MM1. The random binning methods were much faster than the separate CpG method. Larger bins (3:1 ratio) did not perform as well as smaller bins (10:1 ratio), which for MM1 had a high true-positive rate (64.4%) and a lower false-positive rate (46.1%). The naive method identified a large number of associated CpG sites and achieved the highest true-positive rate of all methods (72.4%), but 65.1% of all associated sites were false positives. Associations were biased towards the null for all of the “true” sites which were not identified as significant in the C-C analysis (and therefore not used in the imputation procedure) (Table 3, Web Table 8). Where the associations were strong, there was less evidence of bias (Web Figures 6 and 7, Web Table 9).

**Table 3.** Detailed Analysis of the Performance of the Naive Method for Imputing Smoking Status, Assessed by Comparing the EWAS on the Resulting Data Sets With an EWAS on the Complete Data^a^

MM and Scenario	CpG Sites Identified as Significant in C-C Analysis				CpG Sites Identified as Significant in Analysis on the Complete Data and Not in C-C Analysis				All Other CpG Sites			
	Positive		Negative		Positive		Negative		Positive		Negative	
	Mean β (SE)	Mean Bias (SD)	Mean β (SE)	Mean Bias (SD)	Mean β (SE)	Mean Bias (SD)	Mean β (SE)	Mean Bias (SD)	Mean β (SE)	Mean Bias (SD)	Mean β (SE)	Mean Bias (SD)
MM1^b^												
No. of CpG sites	63.2		106.5		85.0		73.7		235,482.8		246,927.8	
Complete data (“truth”)	0.5641 (0.09207)		−0.5962 (0.09115)		0.5261 (0.09249)		−0.5239 (0.09252)		0.1479 (0.09473)		−0.1468 (0.09578)	
Naive method	0.6187 (0.09473)	0.0546 (0.0541)	−0.6400 (0.09405)	−0.0438 (0.0603)	0.5187 (0.09602)	−0.0074 (0.0505)	−0.5150 (0.09609)	0.0089 (0.0530)	0.1625 (0.09782)	0.0147 (0.0568)	−0.1611 (0.09892)	−0.0143 (0.0559)
MM2^c^												
No. of CpG sites	54.2		92.9		91.7		83.8		235,485.1		246,931.3	
Complete data (“truth”)	0.5562 (0.09214)		−0.6010 (0.09091)		0.5301 (0.09242)		−0.5287 (0.09252)		0.1479 (0.09473)		−0.1468 (0.09578)	
Naive method	0.5722 (0.09424)	0.0160 (0.0530)	−0.6136 (0.09338)	−0.0126 (0.0586)	0.4983 (0.09517)	−0.0318 (0.0479)	−0.4978 (0.09529)	0.0309 (0.0498)	0.1465 (0.09686)	−0.0013 (0.0481)	−0.1466 (0.09794)	0.0002 (0.0485)

Abbreviations: C-C, complete-case; EWAS, epigenome-wide association study; MM, missingness mechanism; SD, standard deviation; SE, standard error.

^a^ Average β coefficient (with average SE) and average bias (with SD) for former smokers specifically for the naive method as compared with the EWAS on the complete data set (*n* = 263). The table shows the results of 10 repeats on the full (unreduced) data set. CpG sites were divided into 3 groups: 1) sites identified as significant in the C-C analysis; 2) sites identified as significant in the complete data set and not in the C-C analysis; and 3) all other sites. We divided the β coefficients into positive (>0) and negative (<0) coefficients according to their value in the EWAS on the complete data set. Web Table 8 shows the equivalent results for current smokers (*n* = 22).

^b^ Missing with probability 75% for males aged 57 years or over.

^c^ Missing with probability 50% for males aged 57 years or over and with probability 12.5% for all remaining persons.

The Wu method had a relatively low false-positive rate (37.3%) but did not perform quite as well as the random binning methods (61.7% true-positive rate) (Table 4, Web Tables 9 and 10, Web Figures 6 and 7). The additional forward-stepwise selection process meant that a much smaller number of sites (<10) were used in the imputation step. The Wu bins method produced results very similar to those of the random bins method, with the smaller bins (10:1) achieving a higher true-positive rate and a lower false-positive rate than the larger bins (3:1).

**Table 4.** Detailed Analysis of the Performance of the Wu Method for Imputing Smoking Status, Assessed by Comparing the EWAS on the Resulting Data Sets With an EWAS on the Complete Data^a^

MM and Scenario	CpG Sites Selected From C-C Analysis				CpG Sites Identified as Significant in Analysis on the Complete Data and Not Selected				All Other CpG Sites			
	Positive		Negative		Positive		Negative		Positive		Negative	
	Mean β (SE)	Mean Bias (SD)	Mean β (SE)	Mean Bias (SD)	Mean β (SE)	Mean Bias (SD)	Mean β (SE)	Mean Bias (SD)	Mean β (SE)	Mean Bias (SD)	Mean β (SE)	Mean Bias (SD)
MM1^b^												
No. of CpG sites^c^	1.4		6.2		134.9		156.5		235,494.7		246,945.3	
Complete data (“truth”)	0.6112 (0.0907)		−0.7925 (0.0857)		0.5465 (0.09223)		−0.5666 (0.09173)		0.1479 (0.09473)		−0.1469 (0.09578)	
Wu method	0.6616 (0.09551)	0.0504 (0.0272)	−0.8456 (0.08873)	−0.0531 (0.0357)	0.5411 (0.09744)	−0.0054 (0.0441)	−0.5743 (0.09622)	−0.0077 (0.0408)	0.1606 (0.09961)	0.0127 (0.0471)	−0.1591 (0.1008)	−0.0122 (0.0462)
MM2^d^												
No. of CpG sites^c^	1.8		6.2		134.4		156.3		235,494.8		246,945.5	
Complete data (“truth”)	0.5735 (0.09118)		−0.7211 (0.08725)		0.5465 (0.09222)		−0.5690 (0.09166)		0.1479 (0.09473)		−0.1469 (0.09578)	
Wu method	0.6313 (0.09471)	0.0578 (0.0513)	−0.7522 (0.09058)	−0.0311 (0.0424)	0.5362 (0.09736)	−0.0103 (0.0482)	−0.5648 (0.09632)	0.0042 (0.0423)	0.1559 (0.09948)	0.0080 (0.0461)	−0.1552 (0.1007)	−0.0083 (0.0461)

Abbreviations: C-C, complete-case; EWAS, epigenome-wide association study; MM, missingness mechanism; SD, standard deviation; SE, standard error.

^a^ Average β coefficient (with average SE) and average bias (with SD) for former smokers specifically for the Wu method as compared with the EWAS on the complete data set (*n* = 263). The table shows the results of 10 repeats on the full (unreduced) data set. CpG sites were divided into 3 groups: 1) sites selected by means of the Bayesian Information Criterion from those identified as significant in the C-C analysis; 2) sites identified as significant in the EWAS on the complete data set which were not selected by the Bayesian Information Criterion; and 3) all other sites. We divided the β coefficients into positive (>0) and negative (<0) coefficients according to their value in the EWAS on the complete data set. Web Table 10 shows the equivalent results for current smokers (*n* = 22).

^b^ Missing with probability 75% for males aged 57 years or over.

^c^ Average number of CpG sites in that group, across the 10 repeats.

^d^ Missing with probability 50% for males aged 57 years or over and with probability 12.5% for all remaining persons.

Results for MM2 were similar to those of MM1, though fewer associated CpG sites were found for most methods, producing correspondingly lower true- and false-positive rates (Table 2).

Results for each method for the 1,000 repeats were very similar to those for the 10 repeats on the full (unreduced) data set and confirmed that the imputation methods involving random binning performed best (Web Table 11, Web Figure 8). Use of 1,000 repeats showed fewer false positives in all of the imputation methods—that is, a lower rate of type 1 errors (Web Table 11). This was particularly true for the random binning methods, which also identified a higher number of true positives (Web Table 11). The C-C, separate CpGs, and random binning methods were unbiased in comparison with the EWAS on the data set with no missingness (Web Tables 12 and 13), but the naive and Wu methods showed bias towards the null for sites not selected for the imputation model—though bias was less obvious since the reduced set of CpGs were deliberately chosen for their strong associations (Web Tables 14–16, Web Figures 9 and 10).

### Application to an EWAS of smoking

The C-C analysis identified 18 CpG sites associated with smoking in the ARIES data set. More associations were identified when smoking status was imputed: The random binning method identified 36, the Wu method identified 29, and the Wu bins method identified 46 (Table 5, Web Table 17). There was a large amount of overlap in the associated CpG sites identified by the 4 methods (Web Table 18), and 62% of all sites identified by at least 1 method were identified in a previous meta-analysis (36) (Table 5, Web Table 19, Web Figure 11). In that meta-analysis, Joehanes et al. (36) examined former smokers versus never smokers and restricted the sites to those that were differentially methylated in current smokers versus never smokers. Note that the meta-analysis was not a gold standard but was an indication of sites found to be related to smoking in other studies. As with the simulation study, the C-C and Wu methods both tended to identify only the strongest associations—they detected fewer significant results, and those results tended to be those with the strongest associations.

**Table 5.** Number of CpG Sites Identified as Associated with Former or Current Smoking in the ARIES Data Set and Their Replication in the Literature^a^

Imputation Method	No. of CpG Sites Identified by the Imputation Method	% of Sites Identified by the Imputation Method That Were Also Reported by Joehanes et al. (36)^b^		% of the 185 Sites Identified After BC in Joehanes et al. (36) That Were Identified by the Imputation Method
		At the 2,568 Sites	At the 185 Sites After BC	
Complete-case	18	94.4	83.3	8.1
Random bins	36	72.2	50.0	9.7
Wu method	29	93.1	82.8	13.0
Wu bins	46	63.0	47.8	11.9
Total unique	60	61.7	43.3	14.0

Abbreviations: ARIES, Accessible Resource for Integrated Epigenomics Studies; BC, Bonferroni correction.

^a^ Shown are the number of CpG sites which were significantly associated with former or current smoking in the ARIES data set, the percentage of these which were replicated at the 2,568 CpG sites reported by Joehanes et al. (36) (current smokers vs. never smokers (false discovery rate < 0.05)), the percentage of those which were replicated at the 185 CpG sites reported by Joehanes et al. (36) after BC, and the percentage of those 185 which were identified by each method.

^b^ A 2016 review of other epigenome-wide association studies of smoking (36).

## DISCUSSION

Using MI to reduce the impact of missing phenotype data can improve the statistical power of EWAS. However, if the MI is carried out naively, bias can result. In our simulation study, the improvement in power and detection of associated sites varied among the MI methods proposed—the optimal methods included using random binning to reduce the number of imputations while keeping bias low. Completely random bins were simpler to implement than those including a subset of CpG sites selected using the Wu method, and they performed just as well in our example. However, if some CpG sites are very strongly related to exposures or covariates, there may be benefits from including them in all imputation bins. The standard error was slightly reduced if larger bins were used, though larger bins also increased the number of falsely identified CpG sites. We have provided R code for the random bins and Wu bins methods in a GitHub repository (37).

The random binning method used a 3:1 or 10:1 ratio of individuals to variables. These ratios are generally accepted (28, 29), though the absolute limit for an imputation model is defined by the number of complete cases in the data set. The naive method resulted in over 150 CpG sites being selected for the imputation model. In the Tsaprouni et al. (22) data set (464 individuals), 150 sites (variables) would be at the limit of the 3:1 ratio of individuals to variables, and this ratio may also be restrictive in other situations. Working at this upper limit may lead to overfitting or models’ failing to fit. In a study that analyzed optimal bin sizes for imputation, though with far fewer variables, Yin et al. (30) found that increasing the bin size improved the imputation quality. However, there is evidence that very large bins (i.e., including many variables in the imputation model) can bias estimates towards the null when imputing an exposure (14), especially when the number of complete cases is small. Imputing for individual sites is effectively creating bins of size 1, and including all sites in a single imputation model (not performed here) is at the other end of the scale (1 bin of ≥480,000 sites), with our bin sizes in the middle. The binning procedure is thus a careful balance between overfitting, bias, and computing time.

CpG sites could be divided into bins based on their gene assignment or distance between base pairs. These methods were considered but not used (Web Appendix 2) because of the wide variety of bin sizes and the risk of collinearity. There are other variable selection methods for MI on high-dimensional data (e.g., a random forest method (38)), but, like the variable selection methods evaluated here (the Wu and naive methods), these will suffer from bias towards the null for sites that are not included in the imputation model. As the illustrative simulation showed (Web Appendix 1), where an association is strongest there will be less evidence of bias, and where an association is weakest the standard errors will be very large, making it hard to distinguish bias from noise. This helps explain why the bias observed here when using the Wu and naive methods was small in comparison with the standard errors.

Inverse probability weighting (IPW) could be used to correct the bias resulting from C-C analyses, by weighting to make the set of complete cases representative (39). In theory, a high number of covariates should not be an issue for IPW; however, it does rely on being able to define a model for missingness accurately. Two IPW methods were implemented (Web Appendix 2). Although IPW was computationally efficient, both IPW methods performed poorly, with large standard errors, in comparison with other methods (Web Tables 7, 8, and 20; Web Figures 4 and 5). In agreement with our results, in general, IPW is unbiased but less efficient than MI (39).

Our EWAS was not equivalent to that of Tsaprouni et al. (22), who adjusted for additional confounders and excluded some probes. The simulation on the Tsaprouni data set was used to illustrate the methods and was deliberately simple, with only 2 covariates used in the MMs and EWAS. In reality, missingness may be a consequence of many covariates, which should all be included in the imputation model. If there were more auxiliary variables giving information on missing smoking status, it is likely that imputation would be improved by including them (i.e., the imputed estimates would have smaller standard errors). However, as more covariates were used (many of which had missingness), the imputation process became slower.

All imputation methods reduced the standard errors and therefore increased detection of associated CpG sites over the C-C analysis. Imputation should be considered whenever missing covariate data limit the sample size for a high-dimensional data set. Such analyses should explore sensitivity to the key assumptions: that the data are missing at random and the imputation model has been correctly specified.

## Supplementary Material

Web Material

## Abbreviations

ALSPAC: Avon Longitudinal Study of Parents and Children
ARIES: Accessible Resource for Integrated Epigenomics Studies
C-C: complete-case
EWAS: epigenome-wide association study(ies)
IPW: inverse probability weighting
MI: multiple imputation
MM: missingness mechanism

## References

[1] Carpenter J, Kenward M Multiple Imputation and Its Application. Hoboken, NJ: John Wiley & Sons, Inc.; 2012.

[2] Sterne JA, White IR, Carlin JB, et al Multiple imputation for missing data in epidemiological and clinical research: potential and pitfalls. BMJ. 2009;338:b2393. 19564179 10.1136/bmj.b2393 PMC2714692

[3] van Buuren S Flexible Imputation of Missing Data. Boca Raton, FL: CRC Press; 2012.

[4] White IR, Carlin JB Bias and efficiency of multiple imputation compared with complete‐case analysis for missing covariate values. Stat Med. 2010;29(28):2920–2931. 20842622 10.1002/sim.3944

[5] Deng Y, Chang C, Ido MS, et al Multiple imputation for general missing data patterns in the presence of high-dimensional data. Sci Rep. 2016;6:21689. 26868061 10.1038/srep21689 PMC4751511

[6] Liao SG, Lin Y, Kang DD, et al Missing value imputation in high-dimensional phenomic data: imputable or not, and how? BMC Bioinformatics. 2014;15:Article 346. 25371041 10.1186/s12859-014-0346-6 PMC4228077

[7] Zhao Y, Long Q Multiple imputation in the presence of high-dimensional data. Stat Methods Med Res. 2016;25(5):2021–2035. 24275026 10.1177/0962280213511027

[8] van Buuren S, Groothuis-Oudshoorn K Mice: multivariate imputation by chained equations in R. J Stat Softw. 2011;45(3):1–67.

[9] Royston P ICE: Stata module for multiple imputation of missing values. https://ideas.repec.org/c/boc/bocode/s446602.html. Published 2006. Revised October 25, 2014. Accessed August 15, 2016.

[10] Klughammer J, Kiesel B, Roetzer T, et al The DNA methylation landscape of glioblastoma disease progression shows extensive heterogeneity in time and space. Nat Med. 2018;24(10):1611–1624. 30150718 10.1038/s41591-018-0156-x PMC6181207

[11] Rizzardi LF, Hickey PF, Rodriguez DiBlasi V, et al Neuronal brain-region-specific DNA methylation and chromatin accessibility are associated with neuropsychiatric trait heritability. Nat Neurosci. 2019;22:307–316. 30643296 10.1038/s41593-018-0297-8 PMC6348048

[12] Küpers LK, Xu X, Jankipersadsing SA, et al DNA methylation mediates the effect of maternal smoking during pregnancy on birthweight of the offspring. Int J Epidemiol. 2015;44(4):1224–1237. 25862628 10.1093/ije/dyv048 PMC4588868

[13] Bartlett JW, Frost C, Carpenter JR Multiple imputation models should incorporate the outcome in the model of interest. Brain. 2011;134(11):e189. 21646332 10.1093/brain/awr062 PMC3212708

[14] Collins LM, Schafer JL, Kam CM A comparison of inclusive and restrictive strategies in modern missing data procedures. Psychol Methods. 2001;6(4):330–351. 11778676

[15] Kenward MG, Carpenter J Multiple imputation: current perspectives. Stat Methods Med Res. 2007;16(3):199–218. 17621468 10.1177/0962280206075304

[16] Moons KG, Donders RA, Stijnen T, et al Using the outcome for imputation of missing predictor values was preferred. J Clin Epidemiol. 2006;59(10):1092–1101. 16980150 10.1016/j.jclinepi.2006.01.009

[17] Spratt M, Carpenter J, Sterne JA, et al Strategies for multiple imputation in longitudinal studies. Am J Epidemiol. 2010;172(4):478–487. 20616200 10.1093/aje/kwq137

[18] Hernán MA, Hernández-Díaz S, Robins JM A structural approach to selection bias. Epidemiology. 2004;15(5):615–625. 15308962 10.1097/01.ede.0000135174.63482.43

[19] Little RJ Regression with missing X’s: a review. J Am Stat Assoc. 1992;87(420):1227–1237.

[20] Wu C, Demerath EW, Pankow JS, et al Imputation of missing covariate values in epigenome-wide analysis of DNA methylation data. Epigenetics. 2016;11(2):132–139. 26890800 10.1080/15592294.2016.1145328 PMC4846117

[21] Illumina, Inc. Infinium® HumanMethylation450 BeadChip. https://www.illumina.com/documents/products/datasheets/datasheet_humanmethylation450.pdf. Accessed October 26, 2016.

[22] Tsaprouni LG, Yang TP, Bell J, et al Cigarette smoking reduces DNA methylation levels at multiple genomic loci but the effect is partially reversible upon cessation. Epigenetics. 2014;9(10):1382–1396. 25424692 10.4161/15592294.2014.969637 PMC4623553

[23] National Center for Biotechnology Information Gene Expression Omnibus [database]. (Accession number GSE50660). http://www.ncbi.nlm.nih.gov/geo/. Accessed July 21, 2016.

[24] Bodner TE What improves with increased missing data imputations? Struct Equ Modeling. 2008;15(4):651–675.

[25] White IR, Royston P, Wood AM Multiple imputation using chained equations: issues and guidance for practice. Stat Med. 2011;30(4):377–399. 21225900 10.1002/sim.4067

[26] von Hippel PT How many imputations do you need? A two-stage calculation using a quadratic rule. Sociol Methods Res. 2018:doi:0049124117747303. 10.1177/0049124117747303 PMC11361408 39211325

[27] Little RJ, Rubin DB Statistical Analysis with Missing Data. Hoboken, NJ: John Wiley & Sons, Inc.; 2014.

[28] Hardt J, Herke M, Leonhart R Auxiliary variables in multiple imputation in regression with missing X: a warning against including too many in small sample research. BMC Med Res Methodol. 2012;12:Article 184. 23216665 10.1186/1471-2288-12-184 PMC3538666

[29] Peduzzi P, Concato J, Kemper E, et al A simulation study of the number of events per variable in logistic regression analysis. J Clin Epidemiol. 1996;49(12):1373–1379. 8970487 10.1016/s0895-4356(96)00236-3

[30] Yin X, Levy D, Willinger C, et al Multiple imputation and analysis for high‐dimensional incomplete proteomics data. Stat Med. 2016;35(8):1315–1326. 26565662 10.1002/sim.6800 PMC4777663

[31] R Core Team *R: A Language and Environment for Statistical Computing* Vienna, Austria: R Foundation for Statistical Computing; 2016.

[32] Boyd A, Golding J, Macleod J, et al Cohort profile: the ‘children of the 90s’—the index offspring of the Avon Longitudinal Study of Parents and Children. Int J Epidemiol. 2013;42(1):111–127. 22507743 10.1093/ije/dys064 PMC3600618

[33] Fraser A, Macdonald-Wallis C, Tilling K, et al Cohort profile: the Avon Longitudinal Study of Parents and Children: ALSPAC mothers cohort. Int J Epidemiol. 2013;42(1):97–110. 22507742 10.1093/ije/dys066 PMC3600619

[34] University of Bristol Avon Longitudinal Study of Parents and Children. Access data and samples. http://www.bristol.ac.uk/alspac/researchers/access/. Accessed July 31, 2019.

[35] Relton CL, Gaunt T, McArdle W, et al Data resource profile: Accessible Resource for Integrated Epigenomic Studies (ARIES). Int J Epidemiol. 2015;44(4):1181–1190. 25991711 10.1093/ije/dyv072 PMC5593097

[36] Joehanes R, Just AC, Marioni RE, et al Epigenetic signatures of cigarette smoking. Circ Cardiovasc Genet. 2016;9(5):436–447. 27651444 10.1161/CIRCGENETICS.116.001506 PMC5267325

[37] Mills HL MethodsMissingCovariateData. https://github.com/harrietlmills/MethodsMissingCovariateData. Accessed July 31, 2019.

[38] Shah AD, Bartlett JW, Carpenter J, et al Comparison of random forest and parametric imputation models for imputing missing data using MICE: a CALIBER study. Am J Epidemiol. 2014;179(6):764–774. 24589914 10.1093/aje/kwt312 PMC3939843

[39] Seaman SR, White IR Review of inverse probability weighting for dealing with missing data. Stat Methods Med Res. 2013;22(3):278–295. 21220355 10.1177/0962280210395740

