# Supplementary material for: Methods for Dealing With Missing Covariate Data in Epigenome-Wide Association Studies
Source: Am J Epidemiol. 2019 Sep 5;188(11):2021–30. doi: 10.1093/aje/kwz186 (PMC6825836; doi:10.1093/aje/kwz186)
Supplement: Web Material [file kwz186_mills_web_material_final.pdf]

WEB MATERIAL

Methods for Dealing with Missing Covariate Data in Epigenome-Wide Association Studies

Harriet L. Mills, Jon Heron, Caroline Relton, Matt Suderman and Kate Tilling

Affiliations: Medical Research Council Integrative Epidemiology Unit, Bristol Medical School, University of Bristol, Bristol, UK (Harriet L Mills, Jon Heron, Caroline Relton, Matt Suderman and Kate Tilling)

Table of Contents:

Web Appendix 1: Simulation Study Exploring Bias

Web Appendix 1.1: Methods

Web Appendix 1.2: Results and discussion

Web Table 1

Web Figure 1

Web Table 2

Web Figure 2

Web Appendix 2: Supplementary Methods

Web Appendix 2.1: Unused methods for imputation

Web Appendix 2.2: IPW Methods

Web Appendix 2.3: Reducing data set to 2000 CpG sites

Web Appendix 2.4: ALSPAC and ARIES variables accessed

Web Table 3

Web Appendix 3: Supplementary Results

Web Appendix 3.1: Demographics of the Tsaprouni data set

Web Table 4

Web Appendix 3.2: Missingness mechanisms

Web Figure 3

Web Appendix 3.3: Computation time – raw results

Web Table 5

Web Appendix 3.4: Results from the 10 repeats on the full (unreduced) data set

Web Table 6-10

Web Figure 4-7

Web Appendix 3.5: Results from the 1000 repeats on the reduced data set

Web Table 11

Web Figure 8

Web Table 12-15

Web Figure 9-10

Web Table 16

Web Appendix 3.6: Results from the ARIES Application

Web Table 17-19

Web Figure 11

Web Appendix 3.7: IPW Results

Web Table 20

References

Web Appendix 1: Simulation study exploring bias

We designed a simulation study to explore the effects of including or excluding variables from the imputation model on the coefficients from the regression model.

1.1 Methods

The data consist of seven continuous outcomes (the equivalent of methylation at different CpG sites in our main example) and one binary exposure (the equivalent of smoking in our main example). We simulate data for 1000 cases. The substantive models are the same as an EWAS – seven separate linear regressions of each outcome (methylation at each CpG site) on exposure (smoking). We simulate the outcomes to have very weak evidence (x2), less weak evidence (x3-x4), moderate evidence (x1, x5, x6) and strong evidence (x7) of an association with the exposure, by varying the amount of variation in each outcome explained by the exposure. The regression coefficient for each outcome on the exposure is 0.2.

First, linear regression is used to estimate the coefficient for the regression of each outcome on the exposure (seven separate regression models, one for each outcome). The exposure is then made missing completely at random (MCAR) with probably 50%. A complete-case (CC) analysis is performed by fitting seven linear regression models (one for each outcome on exposure) in those individuals with exposure measures; because the data are MCAR no bias is expected in the CC. For each simulation, we then used an imputation model to carry out multiple imputation (MI) of the incomplete exposure variable. In each simulation, the imputation model only included those variables from x2-x7 that were significant in the CC analysis for that simulation. We never included x1 in the imputation model, regardless of its P value in the CC analysis. Here, 1000 simulations are run.

Code for this simulation study is provided at

<https://github.com/harrietlmills/MethodsMissingCovariateData>.

1.2 Results and Discussion

Of the seven outcome variables, the coefficient for the association between exposure and x1 is the most biased; x2-x4 the next most, x5-x6 less biased again and x7 is the least biased, Web Table 1 and Web Figure 1. Where the association with the exposure is strongest (x7) there is less evidence of bias, but where the association was weaker (x2-x3) the standard errors were very large, making it hard to distinguish bias from noise. The true value for the coefficient for all outcomes is 0.2.

The mean bias is also calculated after dividing across simulations according to whether or not the variables were included in the imputation. Where the outcome was included in the imputation model for the exposure there was a positive bias, where the variable was not included there was a negative bias, Web Table 2. This is because, in a simulation in which (by chance) the CC estimate for an outcome was large, that outcome tended to be included in the imputation model. Therefore, both the CC estimate and the imputed estimate for that outcome tended to be higher than the true value (0.2), but the imputed estimate tended to be closer to the true value. In a simulation in which (by chance) the CC estimate for an outcome was small, that outcome tended not to be included in the imputation model, and the imputed estimate was more biased than the CC estimate.

Across individual simulated data sets the empirical estimates would be scattered around the true beta, Web Figure 2. For an outcome where the exposure explained little of the variance (e.g. x2), where the estimates of the coefficient therefore have a larger SE, the SE around the estimate will

include the null value in some simulations (shown in blue in Web Figure 2). Hence in these data sets that outcome will be excluded from the imputation model, because of the P value based inclusion rule.

While each individual estimate is an unbiased estimate of the truth, those taken forward for imputation will tend to be a little larger (black, and black diamond on Web Figure 2) and those excluded will be substantially smaller than true beta (blue, and blue diamond on Web Figure 2).

By including an outcome in the imputation model for the exposure, we would expect an unbiased estimate of its association with the exposure, provided the imputation is specified correctly. However, this tendency for being larger than average would persist - though these estimates would be correct for their respective simulations.

When the outcome is excluded from the imputation model but subsequently regressed on the exposure this estimate would *be further downwardly biased* so the final post-imputation estimate across these simulations would be even closer to the null (as depicted by a red diamond in Web Figure 2). This downward bias may be small compared to the more substantial reduction caused by the initial exclusion rule.

Web Table 1. Mean beta and mean bias for the estimated coefficients between exposure and the seven outcomes in the simulation. Recall that variable x1 is never in the imputation model and that in order to be included in the imputation model variables x2-x7 must be significantly associated with exposure in the complete-case analysis.

		**x1** *	**x2**	**x3**	**x4**	**x5**	**x6**	**x7**
**Complete data (no missingness)**	**Mean beta (mean SE)**	0.199<br>(0.079)	0.190<br>(0.316)	0.199<br>(0.158)	0.205<br>(0.158)	0.197<br>(0.079)	0.197<br>(0.079)	0.202<br>(0.040)
**Complete case**	**Mean beta (mean SE)**	0.197<br>(0.113)	0.180<br>(0.449)	0.204<br>(0.225)	0.196<br>(0.225)	0.202<br>(0.112)	0.195<br>(0.112)	0.204<br>(0.056)
	<i>Mean bias (SD)</i>	-0.003<br>(0.081)	-0.009<br>(0.317)	0.005<br>(0.162)	-0.009<br>(0.157)	0.006<br>(0.079)	-0.002<br>(0.082)	0.002<br>(0.040)
**Multiple imputation**	**Mean beta (mean SE)**	0.101<br>(0.099)	0.117<br>(0.395)	0.144<br>(0.201)	0.141<br>(0.202)	0.167<br>(0.106)	0.160<br>(0.105)	0.200<br>(0.057)
	<i>Mean bias (SD)</i>	-0.098<br>(0.084)	-0.073<br>(0.313)	-0.055<br>(0.156)	-0.064<br>(0.150)	-0.030<br>(0.077)	-0.037<br>(0.078)	-0.002<br>(0.041)

* Outcome x1 is never included in the imputation models.

Web Figure 1. Estimated regression coefficients between the exposure and each of the seven outcomes in the simulation, with SE error-bars. *Outcome x1 is never included in the imputation models.

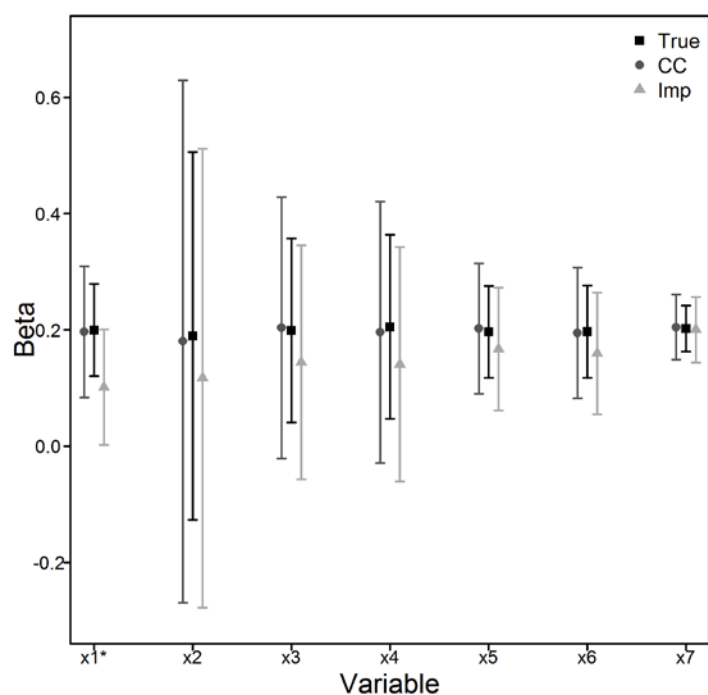

Web Table 2. Bias of estimated regression coefficients between the exposure and each of the seven outcomes in the simulations (mean and SD), split by whether or not they were included in the imputation model. Recall that variable x1 is never in the imputation model and that in order to be included in the imputation model variables x2-x7 must be significantly associated with smoking in the complete-case analysis.

	Mean Bias (SD)						
	x1*	x2	x3	x4	x5	x6	x7
**Variable included in imputation model**							
**CC estimate**	-	0.3112<br>(0.3943)	0.1815<br>(0.1321)	0.1671<br>(0.1187)	0.0553<br>(0.0670)	0.0483<br>(0.0685)	0.0049<br>(0.0386)
**MI estimate**	-	0.2543<br>(0.4143)	0.1624<br>(0.1437)	0.1651<br>(0.1390)	0.0472<br>(0.0731)	0.0437<br>(0.0766)	0.0026<br>(0.0414)
**Variable not included in imputation model**							
**CC estimate**	-0.0027<br>(0.0807)	-0.0321<br>(0.2985)	-0.0268<br>(0.1464)	-0.0381<br>(0.1434)	-0.0345<br>(0.0634)	-0.0384<br>(0.0703)	-0.0543<br>(0.0305)
**MI estimate**	-0.0981<br>(0.0608)	-0.0957<br>(0.2403)	-0.0938<br>(0.1199)	-0.1020<br>(0.1187)	-0.0920<br>(0.0554)	-0.0954<br>(0.0612)	-0.0978<br>(0.0310)

* Outcome x1 is never included in the imputation models.

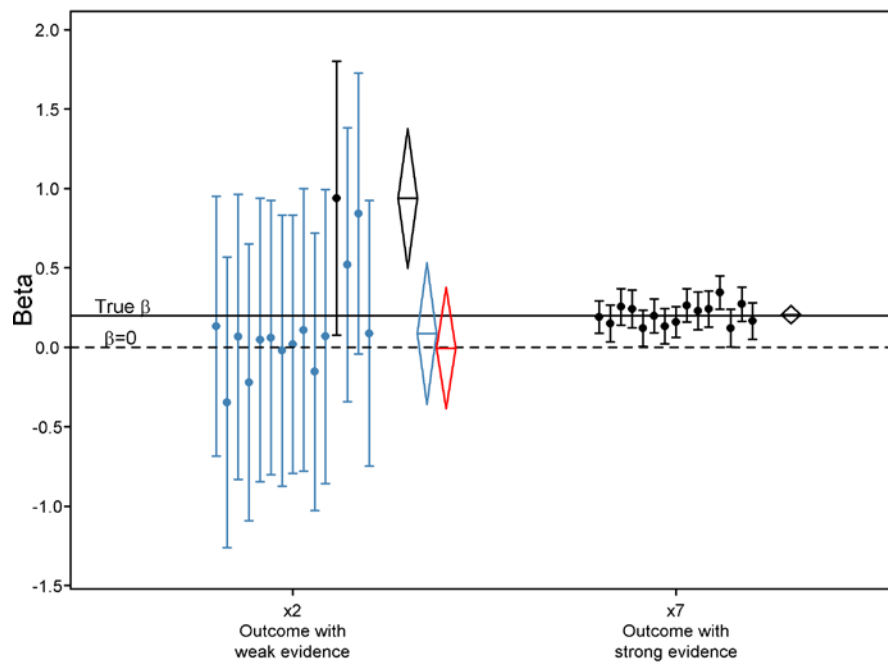

Web Figure 2. Illustration of estimated regression coefficients for the association with exposure, from the complete-case analysis, for a covariate with weak evidence (x2) and a covariate with strong evidence (x7) for 15 randomly chosen simulations. Points show the estimated complete case regression coefficients from individual simulations, with confidence intervals ($1.96 \times SE$) from the estimate. The blue color indicates when the error bar includes the null value, hence when the outcome would be excluded from the imputation model for the exposure. Diamonds show the mean complete case regression coefficient and mean SE for simulations where the outcome would be included in the imputation model for the exposure (black) or excluded (blue). The red diamond shows the mean imputed regression coefficient and mean SE, for those simulations where the outcome was excluded from the imputation.

Web Appendix 2: Supplementary Methods

2.1 Unused methods for imputation

Gene groups

CpGs were clustered according to their gene assignment (using data from Illumina

<ftp://webdata2:webdata2@ussd->

ftp.illumina.com/downloads/productfiles/HumanMethylation450/HumanMethylation450_15017482

[v1-2.csv](#)). Once all clusters were assigned, imputation was performed within the clusters, using all CpGs in the cluster. The result was used for the regressions on every CpG within the cluster.

The problem: Generally, less than 500 CpG sites were assigned to a single gene; however one gene had over 1000 CpGs assigned: to ensure imputation would run (and to not violate the 10:1 and 3:1 proposed ratios) the CpGs could be divided into smaller chunks. Additionally, not every CpG site was assigned a gene, therefore CpGs without an assignment would have had to be clustered using an alternative method (such as *clusterMaker* from the *bumphunter* package in R¹⁻³). These issues made this method unwieldy.

Distance between base pairs

All CpGs were clustered using the *clusterMaker* function in the *bumphunter* package in R¹⁻³ (where a cluster was defined as a sequence of probes such that there were less than 1000 bases between consecutive probes). Imputations were performed in each cluster using all CpGs within the cluster. The resulting imputed data set was used for the regressions on every CpG within the cluster.

The problem: This method gave 168 068 clusters, 67% of which were of size 1 (i.e. a single CpG) and 93% were size 10 or less. 0.09% of clusters were larger than 45 (violating the 10:1 ratio⁴) and 0.007% were larger than 150 (violating the 3:1 ratio⁵). The large number of single clusters meant the imputation was inefficient; also imputation within the much larger groups violated rules about the ratio of variables to samples. Randomly grouping the single clusters for speed and randomly dividing the larger groups would have reproduced the random grouping methods already performed and the method would have been an amalgamation rather than something simple and reproducible. For these reasons, we dropped the method.

2.2 IPW Methods

Two inverse probability weighting methods were used for comparison, one assumed no prior/external knowledge of the missingness mechanism (naive model) and the other assumed complete knowledge (fixed model).

Naive IPW

A binary vector ($R_{smoking}$) was used to indicate whether smoking was missing or present for each individual according to their age and sex. The probability that smoking status was missing for each sample ($p_i^{missing}$) was predicted from the fitted logistic regression model, $R_{smoking} \sim age + sex$ and the analysis model was performed on the complete case data set with weights $w_i =$

$$\frac{1}{(1 - p_i^{missing})}.$$

Fixed IPW

As in naive IPW, but assuming knowledge of the missingness mechanism, so:

MM1: using weights of $w = 1/(1 - 0.75)$ where $sex = male$ and $age \geq 57$ and $w = 1$ otherwise.

MM2: using weights of $w = 1/(1 - 0.5)$ where $sex = male$ and $age \geq 57$ and $w = 1/1 - 0.125$ otherwise.

2.3 Reducing data set to 2000 CpG sites

In order to confirm conclusions by performing more repetitions, the data set was reduced to only 2000 CpG sites (removing 480 739 sites) by keeping all CpG sites identified as associated in the analysis on the complete data set (298 sites), and then keeping the remaining 1702 sites with the highest absolute value of beta (for former smokers). Then 1000 data sets with missingness were generated for both missingness scenarios; and each of these data sets was analysed using the five multiple imputation procedures defined above. Note that the same Bonferroni corrected P value ($0.05/482\ 739$) was used as with the whole data set, because we were not trying to simulate the method on a smaller data set, rather we were trying to repeat the simulation of the high dimensional analysis in an efficient manner.

2.4 ALSPAC and ARIES variables accessed

Web Table 3. Details of the variables accessed from the ALSPAC and ARIES data sets. All missing variables were imputed using every other covariate, including batch effects.

Variable	Question Number	Question/Record	Missingness in ARIES (n=974)	Demographics in ARIES (n=974)		Imputation Method in mice Package*
				Label	Value	
Smoking Status	t5520 and t5560	Derived variable: (Current, Ex and Never) from the questions<br>t5520: Are you currently a smoker?<br>t5560: Have you ever smoked in the past?<br>“Never” (answered “no” to both), “Ex” (answered “yes” to t5560) and “Current” (answered “yes” to t5520)	34.60%	Never	36.30%	polyreg
				Ex	23.60%	
				Current	5.40%	
				Missing	34.60%	
Age of mothers at birth of ALSPAC child	NA		0.20%	Average (yrs)	29.9	norm
Education level	c645a	Derived variable: Mother’s highest educational qualifications	2.10%	CSE	8.20%	polyreg
				Vocational	7.20%	
				O-Level	33.50%	
				A-Level	28.90%	
				Degree	20.20%	
Housing tenure	Derived variable from a006	Derived variable: Home ownership status as reported by the mother early in pregnancy (Mortgaged, Privately rented, Subs rented)	2.70%	Mortgaged	85.90%	polyreg
				Privately rented	5.40%	
				Subsidised rented	6.00%	
				Missing	2.70%	
Social Class	Derived variable from c755 and c765	Derived variable: The highest of the mother and partner's social class, as derived from questions asked of the mother during late pregnancy:<br>0. iv-v (partly skilled occupations and unskilled occupations)<br>1. iiin and iiim (skilled non-manual and manual occupations)	4.60%	0. iv-v	4.00%	polyreg
				1. iiin and iiim	30.40%	
				2. ii	41.40%	
				3. i	19.60%	
				Missing	4.60%	

		2. ii (managerial and technical occupations)<br>3. i (professional occupations)				
Parity	Derived variable from b032	Derived variable: The number of previous pregnancies resulting in either a livebirth or a stillbirth (First, Second, Third, 4 th plus)	3.40%	First	45.50%	polyr
				Second	35.70%	
				Third	11.60%	
				4 th plus	3.80%	
				Missing	3.40%	
Baby birth weight	kz030	Birthweight from obstetric data, birth notification or as recorded by the ALSPAC measurers	2.00%	Average (kg)	3.5kg	norm
Smoking at time of ALSPAC pregnancy	b650 and b659	b650: Have you ever been a smoker?<br>b659: Have you now stopped smoking?<br>Derived variable:<br>“No” if answered “No” to b650 and did not answer b659<br>“Ex” if answered “Yes” to both<br>“Current” if answered “Yes” to b650 and “No” to b659	3.00%	Never	59.90%	polyreg
				Ex	26.20%	
				Current	11.00%	
				Missing	3.00%	
Maternal alcohol consumption	B720, b721 and b722	How often have you drunk alcoholic drinks?<br>b720: Before this pregnancy<br>b721: 1st 3 months of this pregnancy<br>b722: At around the time you first felt the baby move<br>“Non drinkers” (all who answered “never” to b720), “Before 18 weeks” (everyone who said they drank in months 1-3), “After 18 weeks” (everyone who said they drank around the time the baby moved)	3.80%	Non drinkers	32.00%	polyreg
				Before 18 weeks	13.60%	
				After 18 weeks	50.60%	
				Missing	3.80%	

*Methods in *mice* package: Polyreg = Polytomous logistic regression (factor, >= 2 levels); Norm = Bayesian linear regression (numeric); Polyr = Proportional odds model (ordered, >=2 levels).

Web Appendix 3: Supplementary Results

3.1 Demographics of the Tsaprouni data set

Web Table 4. Demographic characteristics and smoking status of the Tsaprouni et al. 2014 data set [Tsaprouni et al. 2014]. The number and percentage of smoking status indicators missing in each of the missingness mechanisms (MM) is shown in the final rows.

	Male	Female	Overall
N (%)	327 (70.5%)	137 (29.5%)	464 (100%)
**Age**			
Mean age (yrs)	54.6	57.3	55.4
Median age (yrs)	55	58	56
**Smoking status**			
% non smokers	37.0%	42.3%	38.6%
% former smokers	58.4%	52.6%	56.7%
% current smokers	4.6%	5.1%	4.7%
**Percentage of smoking missing, for difference missingness mechanisms (MM)**			
10 Reps: N (%) MM1	106.6 (32.6%)	0 (0.0%)	106.6 (23.0%)
10 Reps: N (%) MM2	94.7 (29.0%)	7 (5.1%)	101.7 (21.9%)
1000 Reps: N (%) MM1	107.1 (32.8%)	0.0 (0.0%)	107.1 (23.1%)
1000 Reps: N (%) MM2	94.4 (28.9%)	6.6 (4.8%)	1.0 (21.8%)

3.2 Missingness mechanisms

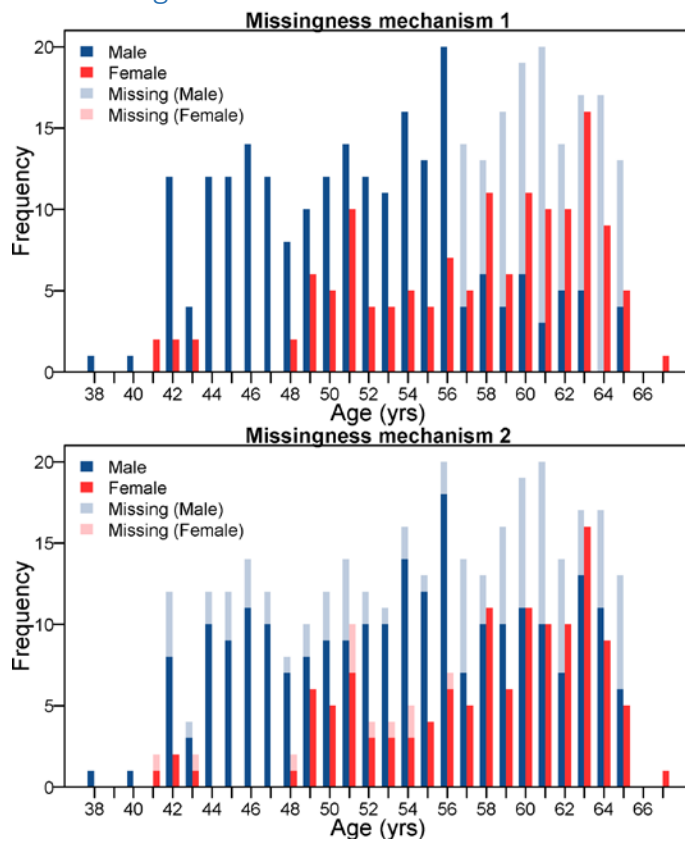

Web Figure 3. An example of the missingness mechanisms explored in the Tsaprouni data set. Missingness mechanism (MM) 1 has a risk of missing smoking status only in males of 57 years old and over; MM2 has missingness across the whole data set, but males of 57 years and over have four times the risk of having missing smoking status.

3.3 Computation time – raw results

Web Table 5. Average time for a single repeat of the analysis on the complete Tsaprouni data set, in hours. Analysis was in R, on a desktop computer with Intel® Core™ i7-6700 CPU @3.40GHz with 16GB RAM. Table 2 in main paper shows the time relative to the method looking at separate CpGs.

	Average Time (hours)
Complete Case	1.1
Separate CpGs	556.0
Random bins (3:1)	287.3
Random bins (10:1)	188.7
Naïve CpGs	38.3
Wu Method	33.1
Wu bins (3:1)	293.0
Wu bins (10:1)	229.2
IPW	1.1
Fixed IPW	556.0

3.4 Results from the 10 repeats on the full (unreduced) data set

Web Table 6. Results from the 10 repeats on the full (unreduced) data set. Average beta (coefficients) and average SE for these beta, for former smokers (N=263), for the group of CpGs found to be associated with former smoking in the analysis on the complete data set (“True”) and every other site (“Other”), for every method, across 10 repeats. The betas were split into positive and negative before averaging, according to their value in the analysis on the complete data set. Average bias was the average across all methods of the difference between the beta compared to the beta from the regression on the complete data set (the “truth”).

	True				Other			
	Positive		Negative		Positive		Negative	
	Mean<br>(Mean SE)	Mean Bias<br>(SD)	Mean<br>(Mean SE)	Mean Bias<br>(SD)	Mean<br>(Mean SE)	Mean Bias<br>(SD)	Mean<br>(Mean SE)	Mean Bias<br>(SD)
**MM1**								
N	136		162		235495		246946	
Complete Data<br>(“truth”)	0.5472<br>(0.0922)		-0.5756<br>(0.0915)		0.1479<br>(0.0947)		-0.1469<br>(0.0958)	
Complete Case	0.5548<br>(0.1036)	0.00761<br>(0.0525)	-0.5967<br>(0.1031)	-0.02114<br>(0.0492)	0.1470<br>(0.1065)	-0.00084<br>(0.0488)	-0.1471<br>(0.1079)	-0.00019<br>(0.0493)
Separate CpGs	0.5659<br>(0.1003)	0.01869<br>(0.0564)	-0.6041<br>(0.0985)	-0.02855<br>(0.0476)	0.1507<br>(0.1046)	0.00278<br>(0.0504)	-0.1498<br>(0.1057)	-0.00289<br>(0.0507)
Random bins<br>(3:1)	0.5456<br>(0.0967)	-0.00166<br>(0.0612)	-0.5812<br>(0.0960)	-0.00561<br>(0.0564)	0.1433<br>(0.0991)	-0.00456<br>(0.0599)	-0.1433<br>(0.1002)	0.00357<br>(0.0603)
Random bins<br>(10:1)	0.5623<br>(0.0993)	0.01510<br>(0.0596)	-0.6031<br>(0.0976)	-0.02755<br>(0.0486)	0.1504<br>(0.1026)	0.00246<br>(0.0517)	-0.1498<br>(0.1036)	-0.00293<br>(0.0521)
Naive Method	0.5593<br>(0.0955)	0.01206<br>(0.0568)	-0.5886<br>(0.0948)	-0.01305<br>(0.0574)	0.1625<br>(0.0978)	0.01466<br>(0.0568)	-0.1612<br>(0.0989)	-0.01431<br>(0.0559)
Wu Method	0.5423<br>(0.0974)	-0.00497<br>(0.0442)	-0.5846<br>(0.0959)	-0.00901<br>(0.0411)	0.1606<br>(0.0996)	0.01274<br>(0.0471)	-0.1591<br>(0.1008)	-0.01220<br>(0.0462)
Wu bins (3:1)	0.5421<br>(0.0957)	-0.00516<br>(0.0531)	-0.5809<br>(0.0949)	-0.00532<br>(0.0529)	0.1476<br>(0.0979)	-0.00031<br>(0.0547)	-0.1472<br>(0.0990)	-0.00030<br>(0.0557)
Wu bins (10:1)	0.5510<br>(0.0967)	0.00381<br>(0.0449)	-0.5927<br>(0.0954)	-0.01712<br>(0.0432)	0.1565<br>(0.0991)	0.00860<br>(0.0474)	-0.1551<br>(0.1002)	-0.00828<br>(0.0482)

Naive IPW	0.5474<br>(0.1528)	0.00019<br>(0.1261)	-0.5554<br>(0.1488)	0.02015<br>(0.1357)	0.1446<br>(0.1601)	-0.00329<br>(0.1378)	-0.1410<br>(0.1620)	0.00588<br>(0.1412)
Fixed IPW	0.5427<br>(0.1246)	-0.00449<br>(0.0806)	-0.5662<br>(0.1209)	0.00936<br>(0.0907)	0.1483<br>(0.1274)	0.00042<br>(0.0857)	-0.1472<br>(0.1290)	-0.00033<br>(0.0875)
**MM2**								
N	136		162		235495		246946	
Complete Data<br>("truth")	0.5472<br>(0.0922)		-0.5756<br>(0.0915)		0.1479<br>(0.0947)		-0.1469<br>(0.0958)	
Complete Case	0.5494<br>(0.1038)	0.00216<br>(0.0519)	-0.5806<br>(0.1028)	-0.00505<br>(0.0463)	0.1477<br>(0.1063)	-0.00019<br>(0.0471)	-0.1480<br>(0.1076)	-0.00116<br>(0.0475)
Separate CpGs	0.5550<br>(0.1003)	0.00779<br>(0.0535)	-0.5880<br>(0.0988)	-0.01245<br>(0.0450)	0.1507<br>(0.1043)	0.00276<br>(0.0488)	-0.1503<br>(0.1054)	-0.00347<br>(0.0491)
Random bins<br>(3:1)	0.5296<br>(0.0957)	-0.01766<br>(0.0604)	-0.5626<br>(0.0951)	0.01293<br>(0.0563)	0.1412<br>(0.0980)	-0.00669<br>(0.0589)	-0.1413<br>(0.0990)	0.00552<br>(0.0590)
Random bins<br>(10:1)	0.5533<br>(0.0991)	0.00606<br>(0.0557)	-0.5875<br>(0.0978)	-0.01195<br>(0.0469)	0.1501<br>(0.1023)	0.00217<br>(0.0504)	-0.1498<br>(0.1034)	-0.00293<br>(0.0508)
Naive Method	0.5291<br>(0.0948)	-0.01808<br>(0.0533)	-0.5607<br>(0.0942)	0.01487<br>(0.0543)	0.1466<br>(0.0969)	-0.00134<br>(0.0481)	-0.1466<br>(0.0979)	0.00023<br>(0.0486)
Wu Method	0.5378<br>(0.0973)	-0.00946<br>(0.0488)	-0.5726<br>(0.0961)	0.00299<br>(0.0427)	0.1559<br>(0.0995)	0.00803<br>(0.0461)	-0.1552<br>(0.1007)	-0.00832<br>(0.0461)
Wu bins (3:1)	0.5208<br>(0.0947)	-0.02641<br>(0.0590)	-0.5576<br>(0.0940)	0.01796<br>(0.0545)	0.1401<br>(0.0967)	-0.00783<br>(0.0556)	-0.1403<br>(0.0977)	0.00651<br>(0.0560)
Wu bins (10:1)	0.5431<br>(0.0966)	-0.00417<br>(0.0481)	-0.5794<br>(0.0956)	-0.00386<br>(0.0438)	0.1514<br>(0.0990)	0.00353<br>(0.0460)	-0.1511<br>(0.1001)	-0.00429<br>(0.0469)
Naive IPW	0.5581<br>(0.1045)	0.01092<br>(0.0554)	-0.5790<br>(0.1016)	-0.00346<br>(0.0513)	0.1518<br>(0.1068)	0.00394<br>(0.0530)	-0.1517<br>(0.1083)	-0.00482<br>(0.0537)
Fixed IPW	0.5455<br>(0.1058)	-0.00176<br>(0.0570)	-0.5696<br>(0.1029)	0.00591<br>(0.0525)	0.1493<br>(0.1082)	0.00142<br>(0.0539)	-0.1494<br>(0.1097)	-0.00254<br>(0.0548)

Web Table 7. Results from the 10 repeats on the full (unreduced) data set. Average beta (coefficients) and average SE for these beta coefficients, for current smokers (N=22), for the group of CpGs to be associated with current smoking in the analysis on the complete data set ("True") and every other site ("Other"), for every method, across 10 repeats. The betas were split into positive and negative before averaging, according to their value in the analysis on the complete data set. Average bias was the average across all methods of the difference between the beta compared to the beta from the regression on the complete data set (the "truth").

	True				Other			
	Positive		Negative		Positive		Negative	
	Mean<br>(Mean SE)	Mean Bias<br>(SD)	Mean<br>(Mean SE)	Mean Bias<br>(SD)	Mean<br>(Mean SE)	Mean Bias<br>(SD)	Mean<br>(Mean SE)	Mean Bias<br>(SD)
**MM1**								
N	113		185		241746		240695	
Complete Data<br>("truth")	0.4475<br>(0.2149)		-0.6726<br>(0.2134)		0.1776<br>(0.2217)		-0.1824<br>(0.2223)	
Complete Case	0.3994<br>(0.2358)	-0.04809<br>(0.1070)	-0.6323<br>(0.2360)	0.04030<br>(0.1169)	0.1627<br>(0.2443)	-0.01497<br>(0.1157)	-0.1657<br>(0.2455)	0.01673<br>(0.1159)
Separate CpGs	0.4078<br>(0.2337)	-0.03971<br>(0.1043)	-0.6351<br>(0.2328)	0.03747<br>(0.1133)	0.1654<br>(0.2424)	-0.01227<br>(0.1160)	-0.1678<br>(0.2432)	0.01463<br>(0.1165)
Random bins<br>(3:1)	0.3558<br>(0.1547)	-0.09174<br>(0.1838)	-0.4415<br>(0.1539)	0.23106<br>(0.2458)	0.1136<br>(0.1589)	-0.06408<br>(0.1564)	-0.1152<br>(0.1593)	0.06721<br>(0.1557)
Random bins<br>(10:1)	0.3908<br>(0.2148)	-0.05672<br>(0.1103)	-0.5712<br>(0.2142)	0.10134<br>(0.1317)	0.1506<br>(0.2226)	-0.02709<br>(0.1149)	-0.1530<br>(0.2231)	0.02943<br>(0.1155)
Naive Method	0.3592<br>(0.1636)	-0.08830<br>(0.1600)	-0.4686<br>(0.1629)	0.20395<br>(0.2179)	0.1098<br>(0.1680)	-0.06785<br>(0.1447)	-0.1127<br>(0.1684)	0.06966<br>(0.1431)
Wu Method	0.3731<br>(0.2287)	-0.07443<br>(0.1041)	-0.5928<br>(0.2289)	0.07973<br>(0.1141)	0.1398<br>(0.2344)	-0.03783<br>(0.0965)	-0.1421<br>(0.2351)	0.04027<br>(0.0952)
Wu bins (3:1)	0.3521<br>(0.1598)	-0.09538<br>(0.1865)	-0.4633<br>(0.1601)	0.20927<br>(0.2265)	0.1165<br>(0.1632)	-0.06115<br>(0.1539)	-0.1186<br>(0.1636)	0.06381<br>(0.1521)
Wu bins (10:1)	0.3586<br>(0.2091)	-0.08892<br>(0.1210)	-0.5460<br>(0.2091)	0.12658<br>(0.1424)	0.1449<br>(0.2146)	-0.03273<br>(0.1131)	-0.1475<br>(0.2151)	0.03492<br>(0.1121)
Naive IPW	0.4873<br>(0.3075)	0.03973<br>(0.3310)	-0.6871<br>(0.3020)	-0.01454<br>(0.3349)	0.1796<br>(0.3024)	0.00195<br>(0.3237)	-0.1847<br>(0.3006)	-0.00226<br>(0.3172)

Fixed IPW	0.4540<br>(0.2805)	0.00653<br>(0.2235)	-0.6715<br>(0.2778)	0.00111<br>(0.2309)	0.1826<br>(0.2743)	0.00500<br>(0.2174)	-0.1864<br>(0.2751)	-0.00400<br>(0.2145)
**MM2**								
N	113		185		241746		240695	
Complete Data<br>("truth")	0.4475<br>(0.2149)		-0.6726<br>(0.2134)		0.1776<br>(0.2217)		-0.1824<br>(0.2223)	
Complete Case	0.4327<br>(0.2511)	-0.01486<br>(0.1408)	-0.6466<br>(0.2500)	0.02601<br>(0.1432)	0.1790<br>(0.2588)	0.00132<br>(0.1384)	-0.1823<br>(0.2599)	0.00012<br>(0.1415)
Separate CpGs	0.4357<br>(0.2460)	-0.01179<br>(0.1375)	-0.6495<br>(0.2467)	0.02305<br>(0.1364)	0.1802<br>(0.2552)	0.00255<br>(0.1389)	-0.1834<br>(0.2562)	-0.00100<br>(0.1422)
Random bins<br>(3:1)	0.3905<br>(0.1727)	-0.05700<br>(0.2008)	-0.4616<br>(0.1721)	0.21098<br>(0.2501)	0.1251<br>(0.1778)	-0.05258<br>(0.1743)	-0.1284<br>(0.1783)	0.05398<br>(0.1720)
Random bins<br>(10:1)	0.4187<br>(0.2318)	-0.02877<br>(0.1282)	-0.5867<br>(0.2321)	0.08587<br>(0.1418)	0.1631<br>(0.2396)	-0.01451<br>(0.1305)	-0.1664<br>(0.2402)	0.01597<br>(0.1320)
Naive Method	0.4180<br>(0.1856)	-0.02951<br>(0.1891)	-0.5011<br>(0.1860)	0.17145<br>(0.2286)	0.1271<br>(0.1905)	-0.05058<br>(0.1749)	-0.1299<br>(0.1910)	0.05249<br>(0.1708)
Wu Method	0.4176<br>(0.2415)	-0.02989<br>(0.1196)	-0.6282<br>(0.2432)	0.04440<br>(0.1273)	0.1589<br>(0.2485)	-0.01878<br>(0.1134)	-0.1624<br>(0.2491)	0.02004<br>(0.1137)
Wu bins (3:1)	0.3957<br>(0.1757)	-0.05186<br>(0.1992)	-0.4866<br>(0.1767)	0.18602<br>(0.2355)	0.1274<br>(0.1796)	-0.05020<br>(0.1761)	-0.1310<br>(0.1801)	0.05139<br>(0.1732)
Wu bins (10:1)	0.4048<br>(0.2289)	-0.04274<br>(0.1270)	-0.5820<br>(0.2302)	0.09058<br>(0.1415)	0.1588<br>(0.2354)	-0.01885<br>(0.1266)	-0.1625<br>(0.2359)	0.01989<br>(0.1258)
Naive IPW	0.4783<br>(0.2724)	0.03073<br>(0.1514)	-0.6758<br>(0.2700)	-0.00317<br>(0.1523)	0.1887<br>(0.2646)	0.01106<br>(0.1505)	-0.1932<br>(0.2662)	-0.01078<br>(0.1528)
Fixed IPW	0.4542<br>(0.2712)	0.00671<br>(0.1550)	-0.6649<br>(0.2697)	0.00770<br>(0.1569)	0.1861<br>(0.2644)	0.00846<br>(0.1559)	-0.1903<br>(0.2670)	-0.00790<br>(0.1595)

Web Table 8. Naive Method Performance Details.

Average beta (average standard error in brackets) and average bias (standard deviation in brackets) for current smokers (N=22) specifically for the Naive method compared to the EWAS on the complete data set. The table shows the results of 10 repeats on the full (unreduced) data set. CpG sites have been divided into three groups: (1) CpG sites found in the CC, (2) CpG sites found in the complete data set and not found in the CC and (3) all other CpG sites. We divide the beta into positive (>0) and negative (<0) according to their value in the EWAS on the complete data set. Table 3 in the main text is the equivalent for former smokers.

	CpG Sites in CC				CpG Sites in Analysis on the Complete Data and Not CC Analysis				All Other CpG Sites			
	Positive		Negative		Positive		Negative		Positive		Negative	
	Mean (SE)	Mean Bias (SD)	Mean (SE)	Mean Bias (SD)	Mean (SE)	Mean Bias (SD)	Mean (SE)	Mean Bias (SD)	Mean (SE)	Mean Bias (SD)	Mean (SE)	Mean Bias (SD)
**Current Smokers**												
**MM1**												
N	66.0		103.7		62.2		96.5		241730.8		240679.8	
Complete Data ("truth")	0.5119 (0.2149)		-0.8222 (0.2121)		0.3614 (0.2157)		-0.4815 (0.2154)		0.1776 (0.2217)		-0.1824 (0.2223)	
Naive Method	0.4012 (0.1627)	-0.1107 (0.1527)	-0.6064 (0.1614)	0.2159 (0.2347)	0.2960 (0.1646)	-0.0653 (0.1622)	-0.3135 (0.1646)	0.1680 (0.1953)	0.1098 (0.1680)	-0.0679 (0.1447)	-0.1127 (0.1684)	0.0697 (0.1431)
**MM2**												
N	53.7		93.4		69.6		105.9		241735.7		240680.7	
Complete Data ("truth")	0.5482 (0.2148)		-0.8409 (0.2118)		0.3694 (0.2155)		-0.4972 (0.2154)		0.1776 (0.2217)		-0.1824 (0.2223)	
Naive Method	0.5047 (0.1798)	-0.0435 (0.1807)	-0.6680 (0.1799)	0.1730 (0.2471)	0.3521 (0.1891)	-0.0172 (0.1969)	-0.3504 (0.1903)	0.1468 (0.2151)	0.1271 (0.1905)	-0.0506 (0.1749)	-0.1299 (0.191)	0.0525 (0.1708)

Web Table 9. Mean standardized beta coefficients for former smokers, grouped by size of the coefficient for the 10 repeats on the full data set. These have been standardized by dividing by the standard errors of the true coefficients. The “Associated” CpGs are the 298 identified by the EWAS on the complete data set; for comparison 298 “Random” CpGs are also included. These coefficients are plotted in Web Figure 6 and Web Figure 7.

	Mean Standardized Beta (SD)								
	≤-5	(-5, -3]	(-3, -2]	(-2, -1]	(-1, 0]	(0, 1]	(1, 2]	(2, 3]	>3
**MM1**									
**Associated, True**	-6.4807<br>(1.6051)	-4.4665<br>(0.3072)	-	-1.6599 (*)	-	-	-	-	5.9383<br>(0.6211)
**Associated, Naive**	-6.6982<br>(1.6323)	-4.3555<br>(0.5096)	-	-1.7152 (*)	-	-	-	-	6.0675<br>(0.8267)
**Associated, Wu**	-6.5734<br>(1.7007)	-4.4653<br>(0.2451)	-	-1.7149 (*)	-	-	-	-	5.8840<br>(0.8035)
**Random 298, True**	-	-3.3974<br>(0.2475)	-2.4223<br>(0.3076)	-1.4986<br>(0.2790)	-0.5422<br>(0.2853)	0.4500<br>(0.2986)	1.4515<br>(0.2754)	2.4518<br>(0.2954)	3.6919<br>(0.6248)
**Random 298, Naive**	-	-3.4793<br>(0.4091)	-2.3509<br>(0.2593)	-1.5547<br>(0.2591)	-0.5259<br>(0.2770)	0.4652<br>(0.3289)	1.4453<br>(0.2860)	2.5052<br>(0.3027)	3.7130<br>(0.6843)
**Random 298, Wu**	-	-3.5199<br>(0.3433)	-2.4360<br>(0.2614)	-1.5018<br>(0.2853)	-0.4377<br>(0.3175)	0.4781<br>(0.2913)	1.4046<br>(0.3126)	2.4702<br>(0.2784)	3.6837<br>(0.6530)
**MM2**									
**Associated, True**	-6.4807<br>(1.6051)	-4.4665<br>(0.3072)	-	-1.6599 (*)	-	-	-	-	5.9383<br>(0.6211)
**Associated, Naive**	-6.3503<br>(1.5564)	-4.3619<br>(0.4126)	-	-1.6133 (*)	-	-	-	2.9196 (*)	5.7624<br>(0.6823)
**Associated, Wu**	-6.4638<br>(1.6445)	-4.5794<br>(0.2509)	-	-1.7756 (*)	-	-	-	2.9316 (*)	5.8566<br>(0.7025)
**Random 298, True**	-	-3.3354<br>(0.3970)	-2.4036<br>(0.2802)	-1.4720<br>(0.3060)	-0.4516<br>(0.2795)	0.4758<br>(0.3073)	1.4820<br>(0.3076)	2.4842<br>(0.2768)	3.4787<br>(0.4484)
**Random 298, Naive**	-	-3.3922<br>(0.3855)	-2.3873<br>(0.3001)	-1.4800<br>(0.3003)	-0.4484<br>(0.3130)	0.4514<br>(0.2840)	1.4085<br>(0.2996)	2.4960<br>(0.2939)	3.5045<br>(0.3881)
**Random 298, Wu**	-	-3.4246<br>(0.3438)	-2.4653<br>(0.3083)	-1.5423<br>(0.2887)	-0.5099<br>(0.2986)	0.4553<br>(0.2624)	1.4288<br>(0.2877)	2.4599<br>(0.2806)	3.4728<br>(0.3562)

*only a single CpG, therefore no SD

Web Table 10. Wu Method Performance Details.

Average beta (average standard error in brackets) and average bias (standard deviation in brackets) for current smokers (N=22) specifically for the Wu method compared to the EWAS on the complete data set. The table shows the results of 10 repeats on the full (unreduced) data set. CpG sites have been divided into three groups: (1) CpG sites selected by Bayesian Inference Criterion (BIC) from those found by the CC, (2) CpG sites found in the EWAS on the complete data set which were not selected by the BIC and (3) all other CpG sites. We divide the beta into positive (>0) and negative (<0) according to their value in the EWAS on the complete data set. Table 4 in the main text is the equivalent for former smokers.

	CpG Sites Selected From CC				CpG Sites in Analysis on the Complete Data And Not Selected				All Other CpG Sites			
	Positive		Negative		Positive		Negative		Positive		Negative	
	Mean<br>(SE)	Mean<br>Bias<br>(SD)	Mean<br>(SE)	Mean<br>Bias<br>(SD)	Mean<br>(SE)	Mean<br>Bias<br>(SD)	Mean<br>(SE)	Mean<br>Bias<br>(SD)	Mean<br>(SE)	Mean<br>Bias<br>(SD)	Mean<br>(SE)	Mean<br>Bias<br>(SD)
**Current Smokers**												
**MM1**												
N	2.0		5.6		111.7		179.7		241745.3		240694.7	
Complete Data ("truth")	0.3695<br>(0.2143)		-1.2293<br>(0.1974)		0.4477<br>(0.2150)		-0.6546<br>(0.2139)		0.1776<br>(0.2217)		-0.1824<br>(0.2223)	
Wu Method	0.3957<br>(0.2247)	0.0262<br>(0.1539)	-1.1767<br>(0.2089)	0.0526<br>(0.0958)	0.3726<br>(0.2288)	-0.0750<br>(0.1036)	-0.5741<br>(0.2295)	0.0805<br>(0.1144)	0.1398<br>(0.2344)	-0.0378<br>(0.0965)	-0.1421<br>(0.2351)	0.0403<br>(0.0952)
**MM2**												
N	2.0		6.0		111.5		179.2		241745.5		240694.8	
Complete Data ("truth")	0.6015<br>(0.2147)		-1.0826<br>(0.2023)		0.4459<br>(0.215)		-0.6587<br>(0.2137)		0.1776<br>(0.2217)		-0.1824<br>(0.2223)	
Wu Method	0.6775<br>(0.2458)	0.0761<br>(0.2519)	-1.0505<br>(0.227)	0.0321<br>(0.1480)	0.4157<br>(0.2416)	-0.0302<br>(0.1186)	-0.6142<br>(0.2437)	0.0445<br>(0.1268)	0.1589<br>(0.2485)	-0.0188<br>(0.1134)	-0.1624<br>(0.2491)	0.0200<br>(0.1137)

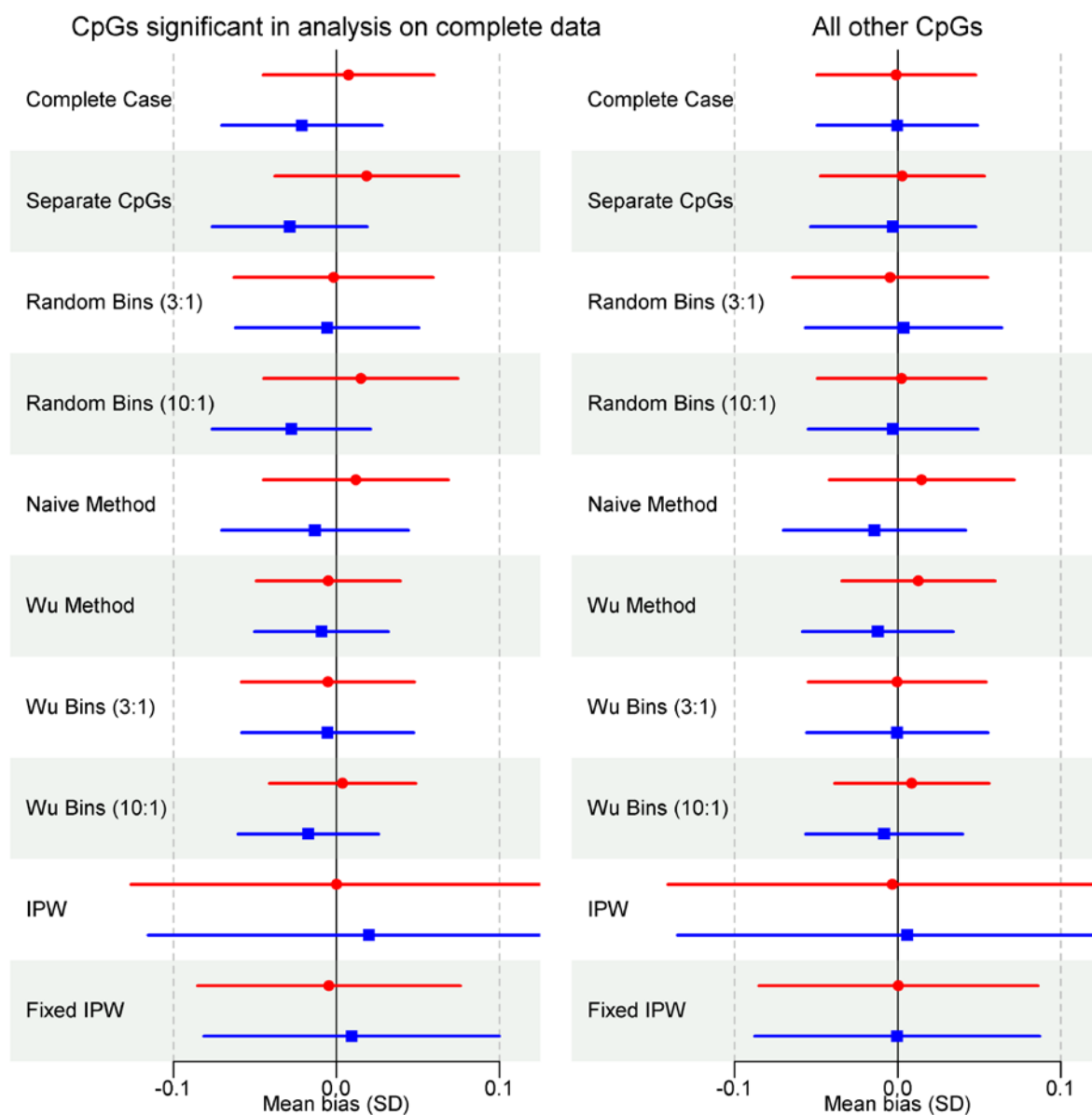

Web Figure 4. The average bias in the coefficients for former smokers (difference between the analysis on the complete data set and that for the imputed data set) for each imputation method (confidence intervals are the standard deviation) and for missingness mechanism 1. (LHS) For the beta (coefficients) of CpGs associated in the full analysis and (RHS) for all other CpG sites. Colors indicate whether the coefficient is positive (red) or negative (blue) in the results of the analysis on the complete data set. This figure illustrates the average bias given in Web Table 6.

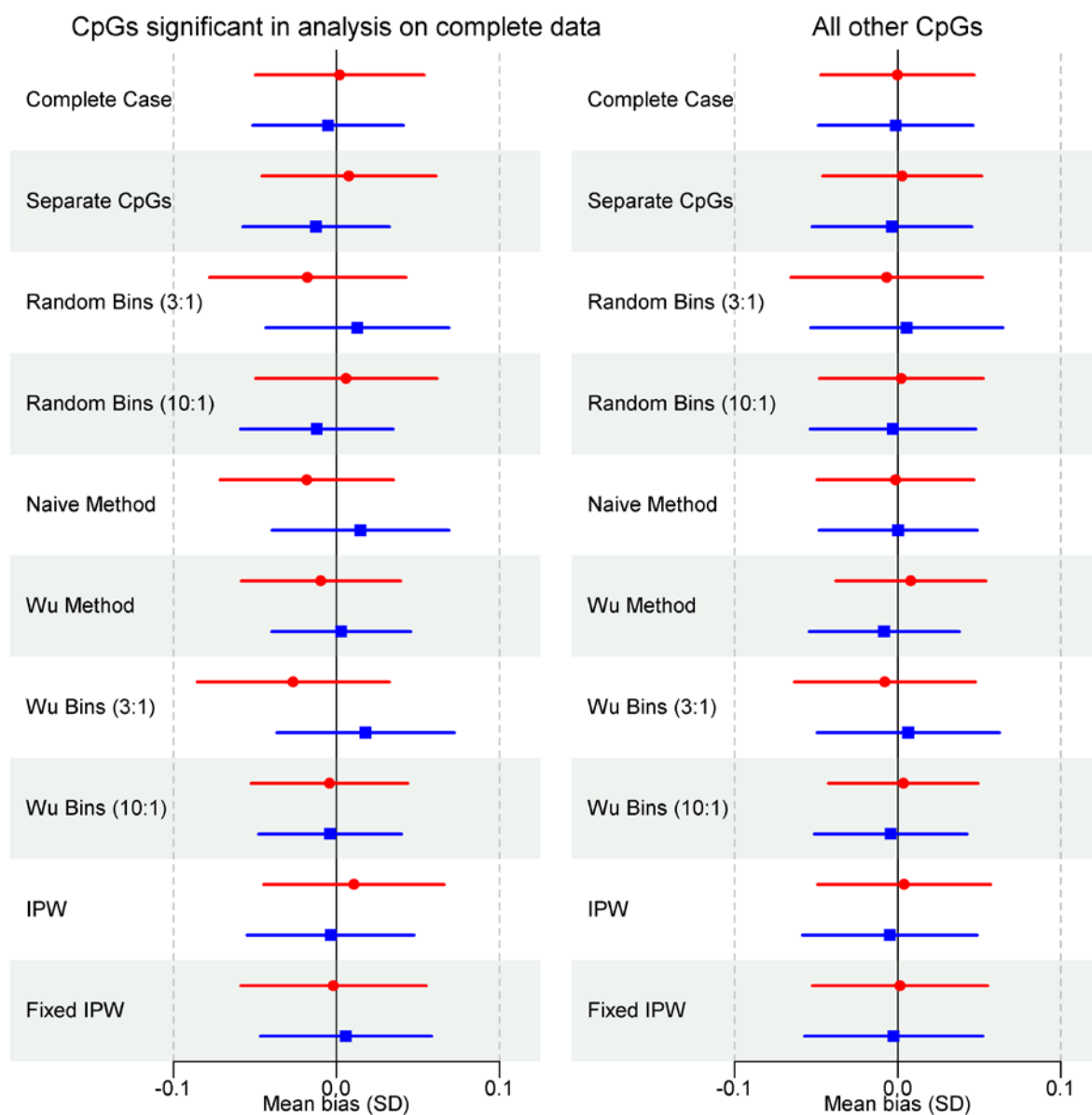

Web Figure 5. The average bias in the coefficients for [former smokers](#) (difference between the analysis on the complete data set and that for the imputed data set) for each imputation method (confidence intervals are the standard deviation) and for [missingness mechanism 2](#). (LHS) For the beta (coefficients) of CpGs associated in the full analysis and (RHS) for all other CpG sites. Colors indicate whether the coefficient is positive (red) or negative (blue) in the results of the analysis on the complete data set. This figure illustrates the average bias' given in Web Table 6.

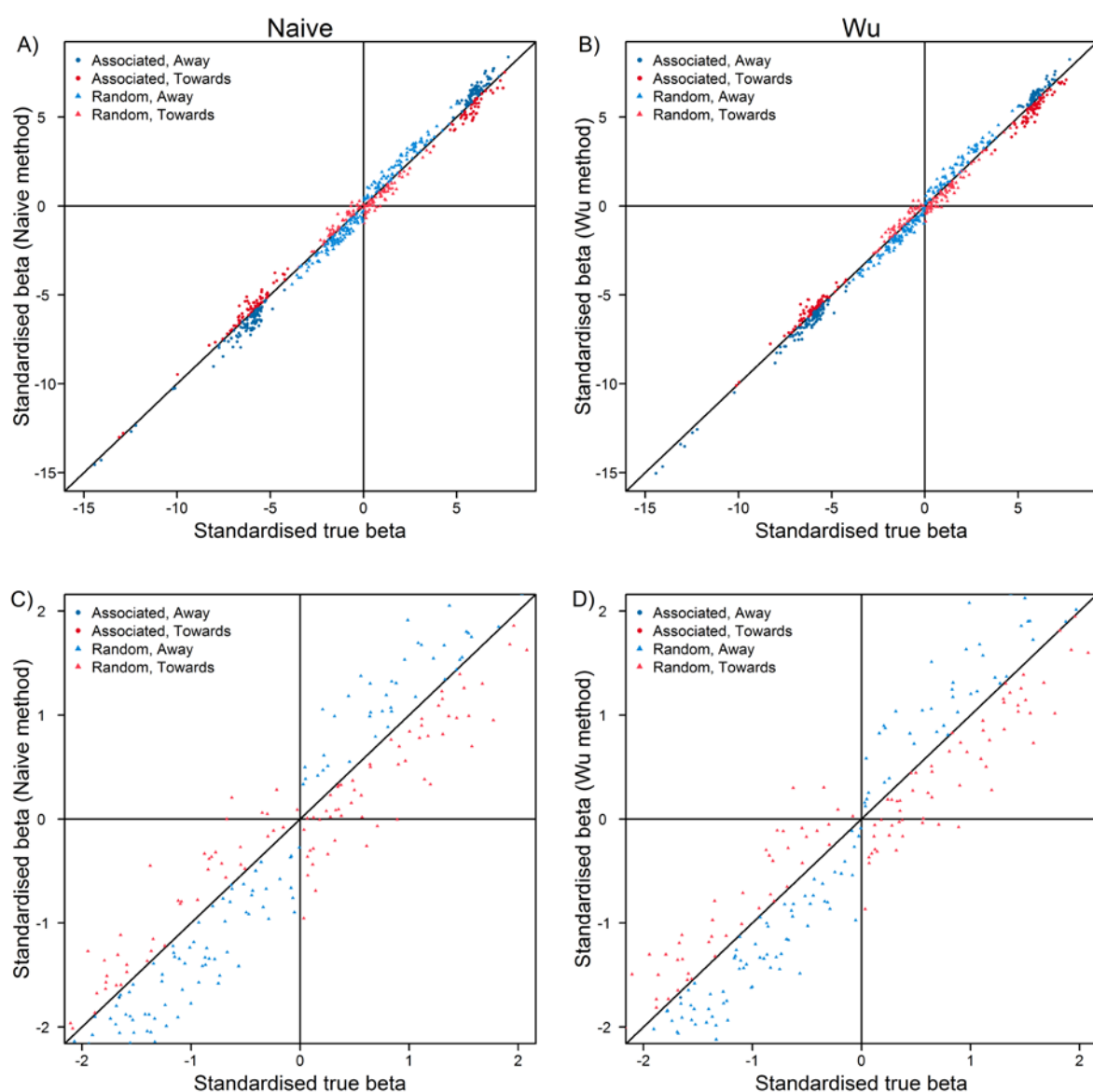

Web Figure 6. Standardized true beta coefficients (for the complete data) against standardized beta coefficients from the data set with missingness, using either the Naive imputation method (A) or the Wu imputation method (B). Panels (C) and (D) show just the central portion of panels (A) and (B). The “Associated” CpGs are the 298 identified by the EWAS on the complete data set (circles); for comparison 298 “Random” CpGs are also included (triangles). Points are colored red if the bias is towards the null, or blue if the bias is away from the null. These coefficients are for former smokers for the 10 repeats on the full data set with missingness model 1. Average values are shown in Web Table 9.

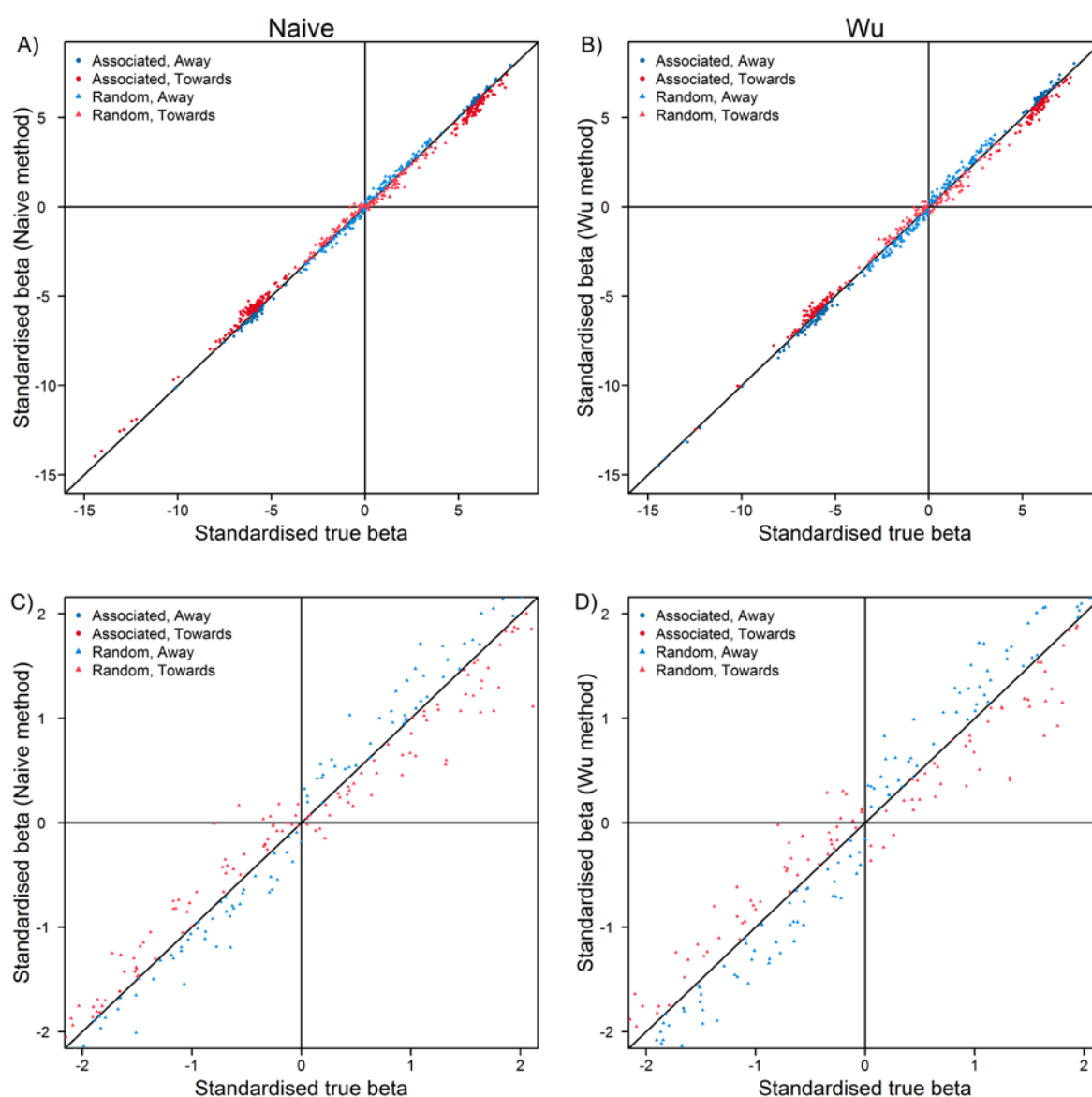

Web Figure 7. Standardized true beta coefficients (for the complete data) against standardized beta coefficients from the data set with missingness, using either the Naive imputation method (A) or the Wu imputation method (B). Panels (C) and (D) show just the central portion of panels (A) and (B). The “Associated” CpGs are the 298 identified by the EWAS on the complete data set (circles); for comparison 298 “Random” CpGs are also included (triangles). Points are colored red if the bias is towards the null, or blue if the bias is away from the null. These coefficients are for former smokers for the 10 repeats on the full data set, with missingness model 2. Average values are shown in Web Table 9.

3.5 Results from the 1000 repeats on the reduced data set (the sensitivity analysis)

Tables below are the equivalent of all main text and supplementary tables presented for the 10 repeats, but the analyses were performed on a reduced data set of 2000 CpG sites (selected as described in the methods). This meant 1000 repeats could be performed.

We note that the CC analysis on the reduced data set did sometimes find a very large number of associated sites. This produced a problem with the Naive method in missingness mechanism 2 because (1) the number of CC sites exceeded the 1:3 or 1:10 ratios of number of variables to number of samples and (2) the “mice” package in R produced an error when the imputation code would have taken an excessive amount of time to complete. Though a tolerance in the imputation function can be increased to avoid this error, we decided not to evaluate these instances (14 in 1000, for missingness mechanism 2 only) because the number of CpG sites selected far exceeded the suggested ratios of variables to samples.

Web Table 11. Results from 1000 repeats on the reduced data set. The number of CpG sites associated with smoking (ex or current) identified in the regression analysis for each method, for both missingness mechanisms (MM). We report the number of these which were also found in the analysis on the complete data (presented with the percentage, i.e. the true positive rate) and the number of those which were not found in the analysis on the complete data set (presented with the percentage of those found which were “incorrect”, i.e. the false positive rate). The mean and SD of the standard errors (for the coefficients for the association of each CpG with being an former smoker) are reported for each method. Note that this is the mean across repeats of the mean and SD of the SE within each repeat.

	MM1				MM2			
	Associated CpGs			SE of coefficients<br>(mean (SD))	Associated CpGs			SE of coefficients<br>(mean (SD))
	Total	No. in<br>complete (%<br>of complete)	No. not in<br>complete (%<br>of total found)		Total	No. in<br>complete (%<br>of complete)	No. not in<br>complete (%<br>of total found)	
Complete Data<br>("truth")	298	-	-	0.0937 (0.0016)	298	-	-	0.0937 (0.0016)
Complete Case	164.8	136.4 (45.8%)	28.4 (15.6%)	0.1054 (0.0031)	153.3	126.5 (42.4%)	26.8 (12.9%)	0.1054 (0.0033)
Separate CpGs	302.8	189.4 (63.5%)	113.4 (34.5%)	0.1019 (0.0027)	269.7	175.7 (58.9%)	94.0 (28.4%)	0.1018 (0.0025)
Random bins (3:1)	434.2	206.1 (69.2%)	228.1 (50.5%)	0.0968 (0.0019)	412.8	199.3 (66.9%)	213.4 (47.6%)	0.0959 (0.0018)
Random bins (10:1)	372.5	204.9 (68.8%)	167.6 (42.6%)	0.0990 (0.0023)	349.8	199.5 (66.9%)	150.3 (37.3%)	0.0989 (0.0022)
Naive Method	620.9	218.5 (73.3%)	402.4 (59.4%)	0.0967 (0.0017)	448.3	195.7 (65.7%)	249.7 (45.8%)	0.0964 (0.0019)
Wu Method	326.5	186.7 (62.6%)	139.8 (36.7%)	0.0984 (0.0021)	296.3	182.8 (61.4%)	113.5 (28.6%)	0.0985 (0.0022)
Wu bins (3:1)	447.6	208.8 (70.1%)	238.7 (50.9%)	0.0967 (0.0018)	417.2	202.8 (68.0%)	214.4 (46.9%)	0.0958 (0.0017)
Wu bins (10:1)	399.2	210.3 (70.6%)	188.9 (43.8%)	0.0978 (0.0021)	373.2	205.9 (69.1%)	167.3 (38.2%)	0.0977 (0.0021)
Naive IPW	74.7	50.3 (16.9%)	24.4 (22.8%)	0.1554 (0.0340)	191.5	139.5 (46.8%)	52.1 (19.8%)	0.1056 (0.0050)
Fixed IPW	81.6	63.1 (21.2%)	18.5 (15.5%)	0.1254 (0.0110)	148.7	116.9 (39.2%)	31.8 (15.2%)	0.1069 (0.0053)

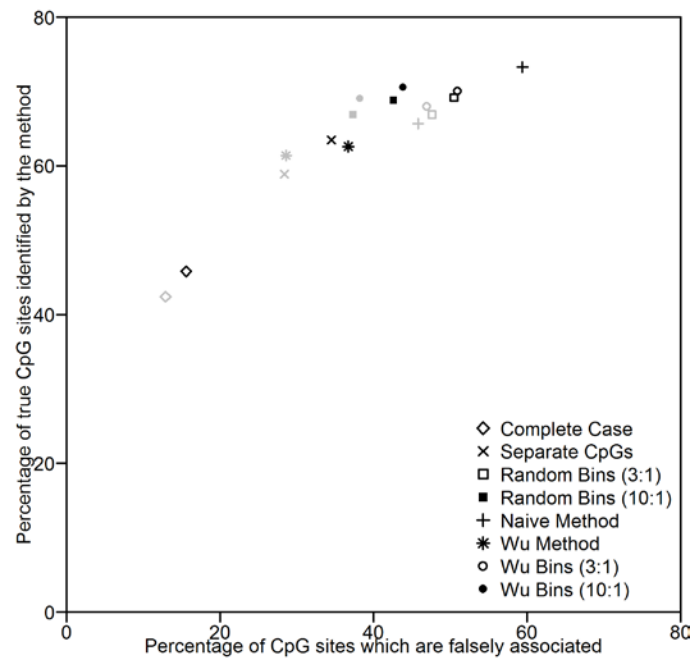

Web Figure 8. Scatter plots of the true positive and false positive percentages identified by the different methods for the 1000 repeats on the reduced data set. Values are listed in Web Table 11. Black symbols are for missingness mechanism 1 and gray are for missingness mechanism 2.

Web Table 12. Results from 1000 repeats on the reduced CpG data set. Average beta (coefficients) and standard deviation for these beta, for former smokers (N=263), for the group of CpGs found to be associated with former smoking in the analysis on the complete data set (“True”) and every other site (“Other”), for every method, across 10 repeats. The betas were split into positive and negative before averaging, according to their value in the analysis on the complete data set. Average bias was the average across all methods of the difference between the beta (coefficients) compared to the beta from the regression on the complete data set (the “truth”).

	True				Other			
	Positive		Negative		Positive		Negative	
	Mean (SD)	Mean Bias (SD)	Mean (SD)	Mean Bias (SD)	Mean (SD)	Mean Bias (SD)	Mean (SD)	Mean Bias (SD)
**MM1**								
N	136		162		890		812	
Complete Data (“truth”)	0.5472<br>(0.0553)		-0.5756<br>(0.1224)		0.4708<br>(0.0224)		-0.4700<br>(0.0224)	
Complete Case	0.5554<br>(0.0850)	0.0082<br>(0.0529)	-0.5995<br>(0.1363)	-0.0239<br>(0.0480)	0.4855<br>(0.0541)	0.0147<br>(0.0481)	-0.4875<br>(0.0535)	-0.0175<br>(0.0477)
Separate CpGs	0.5665<br>(0.0892)	0.0193<br>(0.0560)	-0.6070<br>(0.1383)	-0.0314<br>(0.0470)	0.4943<br>(0.0540)	0.0235<br>(0.0479)	-0.4941<br>(0.0520)	-0.0241<br>(0.0467)
Random bins (3:1)	0.5479<br>(0.0794)	0.0007<br>(0.0528)	-0.5838<br>(0.1353)	-0.0083<br>(0.0507)	0.4748<br>(0.0547)	0.0040<br>(0.0499)	-0.4721<br>(0.0564)	-0.0021<br>(0.0513)
Random bins (10:1)	0.5611<br>(0.0826)	0.0139<br>(0.0505)	-0.5996<br>(0.1356)	-0.0240<br>(0.0441)	0.4905<br>(0.0506)	0.0198<br>(0.0447)	-0.4873<br>(0.0494)	-0.0173<br>(0.0437)
Naive Method	0.5636<br>(0.0802)	0.0164<br>(0.0536)	-0.5933<br>(0.1346)	-0.0177<br>(0.0530)	0.5042<br>(0.0564)	0.0335<br>(0.0522)	-0.4933<br>(0.0565)	-0.0233<br>(0.0517)
Wu Method	0.5450<br>(0.0754)	-0.0023<br>(0.0445)	-0.5874<br>(0.1313)	-0.0118<br>(0.0424)	0.4908<br>(0.0501)	0.0201<br>(0.0454)	-0.4818<br>(0.0469)	-0.0118<br>(0.0421)
Wu bins (3:1)	0.5480<br>(0.0771)	0.0007<br>(0.0510)	-0.5865<br>(0.1356)	-0.0109<br>(0.0499)	0.4777<br>(0.0536)	0.0069<br>(0.0489)	-0.4742<br>(0.0553)	-0.0042<br>(0.0502)
Wu bins (10:1)	0.5545<br>(0.0749)	0.0073<br>(0.0446)	-0.5956<br>(0.1333)	-0.0200<br>(0.0427)	0.4897<br>(0.0480)	0.0189<br>(0.0424)	-0.4854<br>(0.0483)	-0.0154<br>(0.0428)
Naive IPW	0.5581<br>(0.1517)	0.0109<br>(0.1429)	-0.5664<br>(0.1833)	0.0091<br>(0.1380)	0.4782<br>(0.1482)	0.0074<br>(0.1464)	-0.4579<br>(0.1489)	0.0121<br>(0.1469)

Fixed IPW	0.5451<br>(0.1038)	-0.0022<br>(0.0881)	-0.5749<br>(0.1485)	0.0006<br>(0.0850)	0.4681<br>(0.0913)	-0.0027<br>(0.0885)	-0.4681<br>(0.0914)	0.0019<br>(0.0886)
**MM2**								
N	136		162		890		812	
Complete Data<br>("truth")	0.5472<br>(0.0553)		-0.5756<br>(0.1224)		0.4708<br>(0.0224)		-0.4700<br>(0.0224)	
Complete Case	0.5483<br>(0.0787)	0.0011<br>(0.0497)	-0.5848<br>(0.1328)	-0.0092<br>(0.0469)	0.4762<br>(0.0533)	0.0054<br>(0.0478)	-0.4778<br>(0.0533)	-0.0078<br>(0.0481)
Separate CpGs	0.5554<br>(0.0804)	0.0082<br>(0.0497)	-0.5908<br>(0.1346)	-0.0153<br>(0.0447)	0.4825<br>(0.0528)	0.0117<br>(0.0471)	-0.4826<br>(0.0518)	-0.0126<br>(0.0466)
Random bins (3:1)	0.5375<br>(0.0774)	-0.0098<br>(0.0521)	-0.5710<br>(0.1322)	0.0045<br>(0.0501)	0.4648<br>(0.0557)	-0.0060<br>(0.0511)	-0.4642<br>(0.0565)	0.0058<br>(0.0517)
Random bins (10:1)	0.5547<br>(0.0766)	0.0074<br>(0.0462)	-0.5892<br>(0.1325)	-0.0137<br>(0.0421)	0.4824<br>(0.0500)	0.0117<br>(0.0443)	-0.4810<br>(0.0494)	-0.0110<br>(0.0440)
Naive Method	0.5427<br>(0.0769)	-0.0046<br>(0.0522)	-0.5706<br>(0.1304)	0.0050<br>(0.0512)	0.4771<br>(0.0558)	0.0063<br>(0.0516)	-0.4708<br>(0.0557)	-0.0008<br>(0.0513)
Wu Method	0.5415<br>(0.0727)	-0.0058<br>(0.0433)	-0.5775<br>(0.1290)	-0.0019<br>(0.0408)	0.4802<br>(0.0486)	0.0094<br>(0.0439)	-0.4729<br>(0.0468)	-0.0029<br>(0.0419)
Wu bins (3:1)	0.5379<br>(0.0762)	-0.0094<br>(0.0511)	-0.5731<br>(0.1327)	0.0024<br>(0.0489)	0.4661<br>(0.0548)	-0.0046<br>(0.0503)	-0.4651<br>(0.0555)	0.0049<br>(0.0508)
Wu bins (10:1)	0.5501<br>(0.0732)	0.0029<br>(0.0441)	-0.5858<br>(0.1307)	-0.0103<br>(0.0413)	0.4810<br>(0.0488)	0.0102<br>(0.0433)	-0.4789<br>(0.0486)	-0.0089<br>(0.0432)
Naive IPW	0.5571<br>(0.0775)	0.0099<br>(0.0531)	-0.5829<br>(0.1331)	-0.0074<br>(0.0516)	0.4829<br>(0.0580)	0.0121<br>(0.0534)	-0.4777<br>(0.0579)	-0.0077<br>(0.0531)
Fixed IPW	0.5448<br>(0.0779)	-0.0025<br>(0.0552)	-0.5735<br>(0.1328)	0.0021<br>(0.0536)	0.4692<br>(0.0596)	-0.0016<br>(0.0552)	-0.4693<br>(0.0597)	0.0007<br>(0.0554)

Web Table 13. Results from 1000 repeats on the reduced CpG data set. Average beta (coefficients) and standard deviation for these beta, for current smokers (N=22), for the group of CpGs found to be associated with current smoking in the analysis on the complete data set (“True”) and every other site (“Other”), for every method, across 10 repeats. The betas were split into positive and negative before averaging, according to their value in the analysis on the complete data set. Average bias was the average across all methods of the difference between the beta (coefficients) compared to the beta from the regression on the complete data set (the “truth”).

	True				Other			
	Positive		Negative		Positive		Negative	
	Mean (SD)	Mean Bias (SD)	Mean (SD)	Mean Bias (SD)	Mean (SD)	Mean Bias (SD)	Mean (SD)	Mean Bias (SD)
**MM1**								
N	113		185		825		877	
Complete Data (“truth”)	0.4475<br>(0.2700)		-0.6726<br>(0.4729)		0.3386<br>(0.1650)		-0.2968<br>(0.1782)	
Complete Case	0.3898<br>(0.2641)	-0.0577<br>(0.1092)	-0.6260<br>(0.4709)	0.0466<br>(0.1198)	0.2586<br>(0.1727)	-0.0800<br>(0.1159)	-0.2397<br>(0.1989)	0.0571<br>(0.1177)
Separate CpGs	0.3976<br>(0.2693)	-0.0499<br>(0.1067)	-0.6279<br>(0.4706)	0.0447<br>(0.1164)	0.2641<br>(0.1733)	-0.0745<br>(0.1132)	-0.2436<br>(0.1984)	0.0532<br>(0.1155)
Random bins (3:1)	0.3500<br>(0.2288)	-0.0975<br>(0.1793)	-0.4639<br>(0.3438)	0.2087<br>(0.2310)	0.2836<br>(0.1801)	-0.0550<br>(0.1487)	-0.2555<br>(0.1901)	0.0413<br>(0.1622)
Random bins (10:1)	0.3724<br>(0.2386)	-0.0751<br>(0.1152)	-0.5571<br>(0.4078)	0.1154<br>(0.1354)	0.2709<br>(0.1627)	-0.0677<br>(0.1092)	-0.2477<br>(0.1794)	0.0491<br>(0.1128)
Naive Method	0.3567<br>(0.2286)	-0.0908<br>(0.1824)	-0.4546<br>(0.3439)	0.2179<br>(0.2363)	0.2902<br>(0.1763)	-0.0484<br>(0.1489)	-0.2523<br>(0.1843)	0.0445<br>(0.1645)
Wu Method	0.3698<br>(0.2373)	-0.0777<br>(0.1002)	-0.5918<br>(0.4493)	0.0808<br>(0.1124)	0.2518<br>(0.1509)	-0.0868<br>(0.1024)	-0.2332<br>(0.1740)	0.0636<br>(0.1013)
Wu bins (3:1)	0.3425<br>(0.2222)	-0.1051<br>(0.1795)	-0.4695<br>(0.3634)	0.2031<br>(0.2227)	0.2800<br>(0.1764)	-0.0586<br>(0.1465)	-0.2544<br>(0.1865)	0.0424<br>(0.1603)
Wu bins (10:1)	0.3542<br>(0.2249)	-0.0934<br>(0.1228)	-0.5438<br>(0.4047)	0.1288<br>(0.1412)	0.2671<br>(0.1564)	-0.0715<br>(0.1092)	-0.2490<br>(0.1718)	0.0479<br>(0.1147)
Naive IPW	0.4993<br>(0.4425)	0.0518<br>(0.3486)	-0.7078<br>(0.5992)	-0.0352<br>(0.3444)	0.4015<br>(0.4429)	0.0629<br>(0.4039)	-0.3327<br>(0.3970)	-0.0359<br>(0.3501)

Fixed IPW	0.4368<br>(0.3398)	-0.0107<br>(0.2147)	-0.6633<br>(0.5155)	0.0093<br>(0.2132)	0.3249<br>(0.2940)	-0.0137<br>(0.2474)	-0.2867<br>(0.2825)	0.0101<br>(0.2215)
**MM2**								
N	113		185		825		877	
Complete Data<br>("truth")	0.4475<br>(0.2700)		-0.6726<br>(0.4729)		0.3386<br>(0.1650)		-0.2968<br>(0.1782)	
Complete Case	0.4208<br>(0.2846)	-0.0267<br>(0.1270)	-0.6462<br>(0.4776)	0.0263<br>(0.1303)	0.3042<br>(0.2019)	-0.0345<br>(0.1369)	-0.2737<br>(0.2140)	0.0231<br>(0.1294)
Separate CpGs	0.4249<br>(0.2864)	-0.0226<br>(0.1255)	-0.6469<br>(0.4781)	0.0257<br>(0.1261)	0.3075<br>(0.2018)	-0.0311<br>(0.1353)	-0.2756<br>(0.2132)	0.0212<br>(0.1281)
Random bins (3:1)	0.3771<br>(0.2452)	-0.0704<br>(0.1827)	-0.4905<br>(0.3715)	0.1821<br>(0.2226)	0.3064<br>(0.1945)	-0.0323<br>(0.1576)	-0.2685<br>(0.2076)	0.0283<br>(0.1703)
Random bins (10:1)	0.4024<br>(0.2560)	-0.0451<br>(0.1225)	-0.5840<br>(0.4281)	0.0886<br>(0.1335)	0.3040<br>(0.1836)	-0.0346<br>(0.1231)	-0.2702<br>(0.1949)	0.0266<br>(0.1215)
Naive Method	0.3817<br>(0.2434)	-0.0659<br>(0.1798)	-0.4945<br>(0.3761)	0.1781<br>(0.2183)	0.3100<br>(0.1896)	-0.0286<br>(0.1530)	-0.2680<br>(0.2016)	0.0288<br>(0.1650)
Wu Method	0.4088<br>(0.2604)	-0.0387<br>(0.1134)	-0.6197<br>(0.4617)	0.0529<br>(0.1186)	0.2976<br>(0.1783)	-0.0410<br>(0.1180)	-0.2690<br>(0.1924)	0.0278<br>(0.1131)
Wu bins (3:1)	0.3777<br>(0.2421)	-0.0698<br>(0.1796)	-0.5037<br>(0.3935)	0.1689<br>(0.2127)	0.3047<br>(0.1916)	-0.0339<br>(0.1550)	-0.2693<br>(0.2055)	0.0275<br>(0.1675)
Wu bins (10:1)	0.3899<br>(0.2458)	-0.0576<br>(0.1223)	-0.5770<br>(0.4292)	0.0956<br>(0.1338)	0.2961<br>(0.1748)	-0.0426<br>(0.1186)	-0.2675<br>(0.1883)	0.0293<br>(0.1191)
Naive IPW	0.4657<br>(0.3080)	0.0181<br>(0.1501)	-0.6741<br>(0.4936)	-0.0015<br>(0.1493)	0.3607<br>(0.2403)	0.0221<br>(0.1698)	-0.3070<br>(0.2347)	-0.0102<br>(0.1524)
Fixed IPW	0.4463<br>(0.3077)	-0.0012<br>(0.1528)	-0.6673<br>(0.4921)	0.0052<br>(0.1526)	0.3394<br>(0.2378)	0.0008<br>(0.1724)	-0.2984<br>(0.2368)	-0.0016<br>(0.1559)

Web Table 14. Results from the 1000 repeats on the reduced CpG data set. Average beta (coefficients) and average bias for former and current smokers specifically for the Naive method compared to the analysis on the complete data set (standard deviation in brackets). N=263 and N=22 for ex and current smokers respectively. CpG sites have been grouped into three groups: (1) CpG sites found in the CC, (2) CpG sites found in the analysis on the complete data set and not found in the CC analysis and (3) all other CpG sites. We divide the beta into positive (>0) and negative (<0) according to their value in the analysis on the complete data set.

	CpG Sites in CC				CpG Sites in Analysis on the Complete Data and Not CC Analysis				All Other CpG Sites			
	Positive		Negative		Positive		Negative		Positive		Negative	
	Mean (SD)	Mean Bias (SD)	Mean (SD)	Mean Bias (SD)	Mean (SD)	Mean Bias (SD)	Mean (SD)	Mean Bias (SD)	Mean (SD)	Mean Bias (SD)	Mean (SD)	Mean Bias (SD)
**Former Smokers**												
**MM1**												
N	60.0		104.8		87.2		74.4		878.8		794.8	
Complete Data ("truth")	0.5689 (0.0548)		-0.5979 (0.1388)		0.5269 (0.0472)		-0.5260 (0.0605)		0.4703 (0.0220)		-0.4694 (0.0219)	
Naive Method	0.6207 (0.0542)	0.0519 (0.0507)	-0.6432 (0.1365)	-0.0454 (0.0550)	0.5283 (0.0698)	0.0015 (0.0501)	-0.5234 (0.0737)	0.0027 (0.0486)	0.5031 (0.0556)	0.0327 (0.0519)	-0.4911 (0.0545)	-0.0217 (0.0506)
**MM2**												
N	49.6		87.6		95.2		85.5		881.1		800.9	
Complete Data ("truth")	0.5689 (0.0616)		-0.6102 (0.1485)		0.5312 (0.0464)		-0.5298 (0.0565)		0.4705 (0.0222)		-0.4696 (0.0221)	
Naive Method	0.5883 (0.0627)	0.0194 (0.0518)	-0.6194 (0.1422)	-0.0092 (0.0546)	0.5100 (0.0667)	-0.0212 (0.0487)	-0.5067 (0.0705)	0.0230 (0.0489)	0.4710 (0.0545)	0.0005 (0.0506)	-0.4654 (0.0545)	0.0042 (0.0505)
**Current-Smokers**												
**MM1**												
N	62.1		102.7		64.4		97.2		811.5		862.1	
Complete Data ("truth")	0.5080 (0.2622)		-0.8234 (0.4970)		0.3683 (0.2437)		-0.4744 (0.3404)		0.3385 (0.1647)		-0.2947 (0.1767)	

Naive Method	0.3892<br>(0.2314)	-0.1188<br>(0.1678)	-0.5908<br>(0.3256)	0.2326<br>(0.2489)	0.3046<br>(0.2225)	-0.0637<br>(0.1857)	-0.2971<br>(0.2737)	0.1773<br>(0.2168)	0.2907<br>(0.1752)	-0.0477<br>(0.1488)	-0.2504<br>(0.1838)	0.0443<br>(0.1646)
**MM2**												
N	48.0		89.3		73.6		107.1		816.4		865.6	
Complete Data<br>("truth")	0.5280<br>(0.2724)		-0.8505<br>(0.5199)		0.3851<br>(0.2447)		-0.4930<br>(0.3385)		0.3384<br>(0.1645)		-0.2957<br>(0.1772)	
Naive Method	0.4475<br>(0.2500)	-0.0804<br>(0.1710)	-0.6663<br>(0.3882)	0.1842<br>(0.2290)	0.3382<br>(0.2343)	-0.0469<br>(0.1866)	-0.3444<br>(0.3031)	0.1486<br>(0.2051)	0.3089<br>(0.1882)	-0.0294<br>(0.1551)	-0.2656<br>(0.2026)	0.0301<br>(0.1664)

Web Table 15. Results from the 1000 repeats on the reduced CpG data set. Average beta (coefficients) and average bias for former and current smokers specifically for the Wu method compared to the analysis on the complete data set (standard deviation in brackets). N=263 and N=22 for ex and current smokers respectively. CpG sites have been grouped into three groups: (1) CpG sites selected by BIC from those found by the CC, (2) CpG sites found in the analysis on the complete data set which were not selected by the BIC and (3) all other CpG sites. We divide the beta into positive (>0) and negative (<0) according to their value in the analysis on the complete data set.

	CpG Sites Selected From CC				CpG Sites in Analysis on the Complete Data and Not Selected				All Other CpG Sites			
	Positive		Negative		Positive		Negative		Positive		Negative	
	Mean (SD)	Mean Bias (SD)	Mean (SD)	Mean Bias (SD)	Mean (SD)	Mean Bias (SD)	Mean (SD)	Mean Bias (SD)	Mean (SD)	Mean Bias (SD)	Mean (SD)	Mean Bias (SD)
**Former Smokers**												
**MM1**												
N	1.4		5.5		135.0		157.1		889.6		811.3	
Complete Data ("truth")	0.5962 (0.0796)		-0.7780 (0.2431)		0.5466 (0.0545)		-0.5681 (0.1086)		0.4708 (0.0223)		-0.4700 (0.0224)	
Wu Method	0.6600 (0.0737)	0.0638 (0.0361)	-0.8334 (0.2325)	-0.0554 (0.0372)	0.5440 (0.0745)	-0.0026 (0.0444)	-0.5787 (0.1169)	-0.0106 (0.0421)	0.4908 (0.0500)	0.0200 (0.0454)	-0.4817 (0.0468)	-0.0118 (0.0420)
**MM2**												
N	1.8		5.8		134.5		156.6		889.7		811.7	
Complete Data ("truth")	0.5884 (0.0849)		-0.7412 (0.2581)		0.5466 (0.0544)		-0.5693 (0.1092)		0.4708 (0.0223)		-0.4700 (0.0224)	
Wu Method	0.6407 (0.0847)	0.0523 (0.0406)	-0.7762 (0.2553)	-0.0350 (0.0388)	0.5403 (0.0716)	-0.0063 (0.0430)	-0.5702 (0.1153)	-0.0009 (0.0406)	0.4801 (0.0486)	0.0094 (0.0439)	-0.4728 (0.0468)	-0.0028 (0.0419)
**Current Smokers**												
**MM1**												
N	2.0		4.9		111.9		180.2		824.1		876.9	
Complete Data ("truth")	0.3604 (0.2576)		-1.2185 (0.7836)		0.4470 (0.2687)		-0.6575 (0.4509)		0.3388 (0.1649)		-0.2968 (0.1781)	

Wu<br>Method	0.4196<br>(0.1830)	0.0592<br>(0.1400)	-1.1482<br>(0.7542)	0.0703<br>(0.0869)	0.3686<br>(0.2372)	-0.0784<br>(0.0996)	-0.5764<br>(0.4280)	0.0811<br>(0.1130)	0.2517<br>(0.1509)	-0.0871<br>(0.1021)	-0.2331<br>(0.1740)	0.0637<br>(0.1013)
**MM2**												
N	2.1		5.5		111.4		179.7		824.6		876.8	
Complete<br>Data<br>("truth")	0.4330<br>(0.2628)		-1.1294<br>(0.7774)		0.4470<br>(0.2688)		-0.6584<br>(0.4518)		0.3387<br>(0.1649)		-0.2968<br>(0.1781)	
Wu<br>Method	0.4610<br>(0.2539)	0.0280<br>(0.1418)	-1.0933<br>(0.7573)	0.0361<br>(0.1158)	0.4075<br>(0.2602)	-0.0394<br>(0.1129)	-0.6051<br>(0.4417)	0.0533<br>(0.1187)	0.2976<br>(0.1783)	-0.0411<br>(0.1180)	-0.2690<br>(0.1924)	0.0278<br>(0.1131)

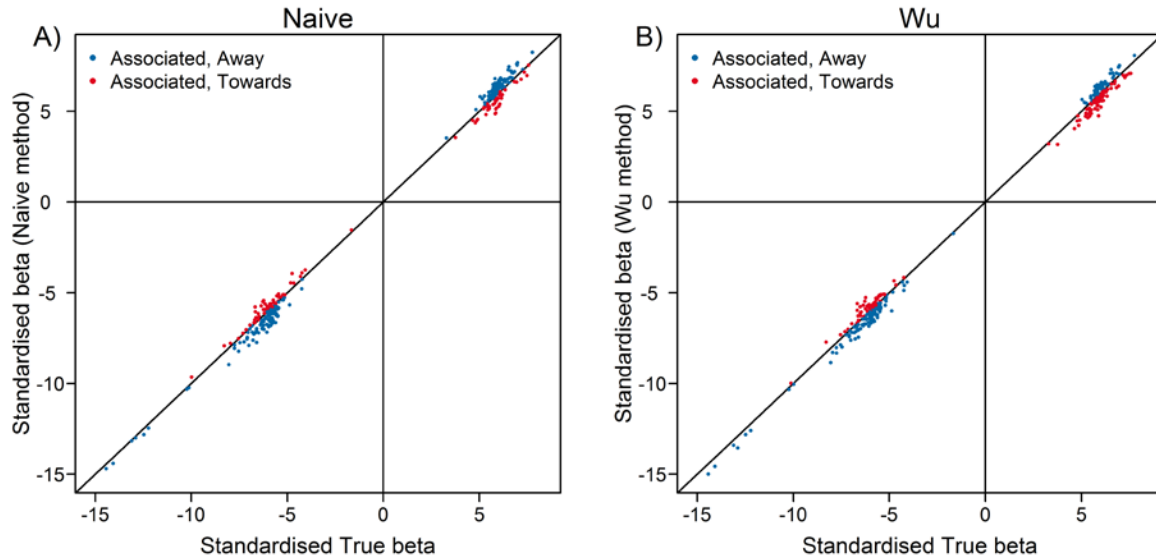

Web Figure 9. Standardized true beta coefficients (for the complete data) against standardized beta coefficients from the data set with missingness, using either the Naive imputation method (A) or the Wu imputation method (B). Points are colored red if the bias is towards the null, or blue if the bias is away from the null. These coefficients are for former smokers for the 1000 repeats on the reduced data set, with missingness model 1. Average values are shown in Web Table 16.

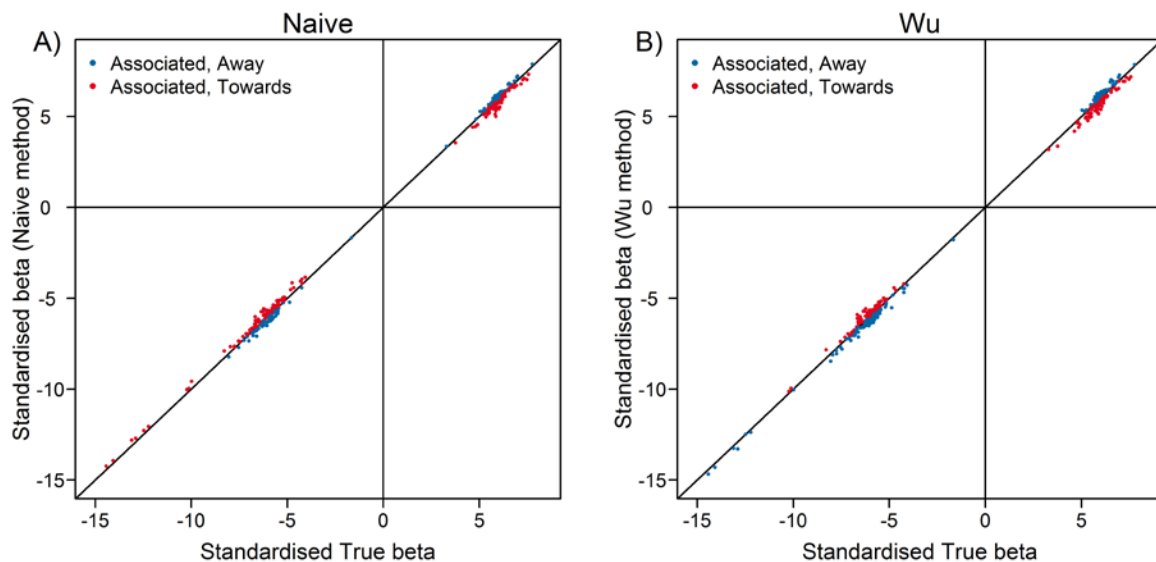

Web Figure 10. Standardized true beta coefficients (for the complete data) against standardized beta coefficients from the data set with missingness, using either the Naive imputation method (A) or the Wu imputation method (B). Points are colored red if the bias is towards the null, or blue if the bias is away from the null. These coefficients are for former smokers for the 1000 repeats on the reduced data set, with missingness model 2. Average values are shown in Web Table 16.

Web Table 16. Mean standardized beta coefficients for former smokers, grouped by size of the coefficient for the 1000 repeats on the reduced data set. These have been standardized by dividing by the standard errors of the true coefficients. The “Associated” CpGs are the 298 identified by the EWAS on the complete data set. These coefficients are plotted in Web Figure 9 and Web Figure 10.

	Mean Standardized Beta (SD)								
	≤-5	(-5, -3]	(-3, -2]	(-2, -1]	(-1, 0]	(0, 1]	(1, 2]	(2, 3]	>3
**MM1**									
**Associated, “Truth”**	-6.4807 (1.6051)	-4.4665 (0.3072)	-	-1.6599 (*)	-	-	-	-	5.9383 (0.6211)
**Associated, Naive**	-6.6855 (1.6382)	-4.2130 (0.3499)	-	-1.5519 (*)	-	-	-	-	6.1143 (0.7691)
**Associated, Wu**	-6.6013 (1.6929)	-4.5473 (0.2684)	-	-1.7324 (*)	-	-	-	-	5.9131 (0.7751)
**MM2**									
**Associated, “Truth”**	-6.4807 (1.6051)	-4.4665 (0.3072)	-	-1.6599 (*)	-	-	-	-	5.9383 (0.6211)
**Associated, Naive**	-6.4513 (1.5778)	-4.4041 (0.4063)	-	-1.6727 (*)	-	-	-	-	5.8879 (0.6640)
**Associated, Wu**	-6.4987 (1.6469)	-4.5442 (0.2547)	-	-1.7818 (*)	-	-	-	-	5.8752 (0.7006)

*only a single CpG, therefore no SD

3.6 Results from the ARIES Application

Web Table 17. Number of CpGs identified as associated with smoking (former or current) in the ARIES data set, using the complete-case analysis and three imputation methods. Reported are the number of CpGs which were associated and the actual time taken on BlueCrystal to run the analyses.

	Number of CpG Sites Identified	Hours to Run
Complete Case	18	3
Random Bins	36	3950
Wu Method	29	121
Wu Bins	46	3836

Web Table 18. The total number of overlapping CpG sites between methods.

Number Overlap	Complete Case	Random Bins	Wu Method	Wu Bins
Complete Case	-	16	18	18
Random Bins	16	-	21	25
Wu Method	18	21	-	25
Wu Bins	18	25	25	-

Web Table 19. The P values for every CpG sites identified in the four methods used in the ARIES application (complete-case analysis and three different imputation methods). Those P values in **bold, red** text are within our Bonferroni threshold of 1.036×10^{-7} .⁷ We check for replication in a meta-analysis of results from 16 cohorts (Joehanes et al.⁶).

CpG site	Complete Case	Random Bins	Wu Method	Wu Bins	Replicated in Joehanes et al.	
					Yes/No	P value
cg05575921	**2.52E-65**	**4.41E-54**	**1.57E-43**	**6.21E-47**	Yes	8.20E-27
cg18146737	**8.76E-11**	**1.58E-10**	**1.30E-11**	**1.49E-10**	Yes	1.00E-05
cg21566642	**1.45E-35**	**2.88E-42**	**2.48E-52**	**2.67E-37**	Yes	1.00E-25
cg27262054	**1.60E-08**	9.44E-07	**3.04E-09**	**1.52E-09**	No	
cg04885881	**2.07E-09**	**7.52E-15**	**8.54E-11**	**2.59E-09**	Yes	5.20E-13
cg09935388	**3.34E-20**	**1.03E-15**	**1.43E-22**	**1.77E-20**	Yes	6.70E-11
cg12876356	**7.28E-08**	2.02E-06	**1.87E-10**	**4.01E-10**	Yes	4.70E-09
cg25189904	**2.33E-12**	**5.62E-13**	**2.68E-14**	**2.39E-14**	Yes	3.50E-40
cg01940273	**1.47E-27**	**7.84E-39**	**1.74E-39**	**7.51E-36**	Yes	9.60E-73
cg03329539	**3.79E-12**	**1.65E-11**	**4.13E-16**	**1.68E-13**	Yes	1.90E-11
cg05951221	**1.14E-27**	**1.33E-35**	**3.43E-42**	**1.33E-44**	Yes	1.10E-27
cg03991871	**1.36E-13**	**1.94E-15**	**9.27E-16**	**1.53E-19**	Yes	1.50E-09
cg12806681	**1.30E-10**	**4.32E-10**	**3.07E-10**	**4.14E-13**	Yes	1.50E-08
cg23576855	**3.37E-17**	**4.90E-14**	**5.55E-23**	**4.56E-23**	Yes	6.10E-13
cg25648203	**3.91E-08**	**8.07E-10**	**4.59E-13**	**1.08E-12**	Yes	6.90E-08
cg06126421	**1.26E-25**	**3.67E-21**	**1.64E-30**	**1.78E-25**	Yes	3.00E-18
cg02451831	**7.08E-08**	**1.03E-07**	**5.12E-10**	**1.68E-09**	Yes	0.001

cg03636183	**1.30E-20**	**1.48E-28**	**1.90E-28**	**5.00E-27**	Yes	8.90E-25
cg14817490	5.35E-07	**4.59E-08**	1.25E-07	1.95E-06	Yes	5.30E-07
cg25953130	1.12E-04	**4.27E-09**	6.08E-07	**2.68E-08**	Yes	8.10E-06
cg19859270	4.38E-07	**6.61E-09**	3.10E-06	1.43E-07	Yes	3.10E-11
cg02599587	3.05E-05	**1.23E-08**	1.17E-04	**7.90E-10**	No	
cg16967296	2.32E-02	**7.70E-08**	3.43E-02	3.92E-04	No	
cg14712058	1.91E-05	**4.55E-08**	7.21E-07	**1.57E-09**	No	
cg15342087	1.58E-06	**9.94E-08**	**1.36E-10**	**1.78E-09**	Yes	1.60E-12
cg21733098	7.98E-06	**4.98E-09**	**8.16E-09**	1.28E-07	Yes	6.70E-05
cg09099830	3.50E-06	**8.17E-08**	1.49E-07	**1.90E-08**	Yes	4.60E-05
cg13845982	1.52E-07	**8.89E-12**	5.88E-05	**1.14E-10**	No	
cg21161138	4.84E-07	**3.76E-10**	**1.48E-10**	**1.39E-10**	Yes	1.60E-14
cg08154348	4.83E-03	**1.68E-08**	3.49E-01	1.54E-02	No	
cg03702413	7.70E-07	**9.96E-08**	2.00E-04	1.13E-06	No	
cg00501876	3.32E-05	**6.03E-08**	2.24E-07	1.54E-07	Yes	0.0011
cg05411171	9.84E-06	**9.28E-10**	2.20E-03	3.49E-05	No	
cg26703534	8.34E-07	**6.51E-09**	**1.44E-14**	**1.10E-12**	No	
cg16497398	2.46E-05	**2.83E-08**	2.06E-04	7.41E-07	No	
cg21960364	1.11E-05	**5.59E-09**	5.99E-03	2.09E-06	Yes	0.0047
cg13716167	2.93E-03	**5.13E-08**	1.12E-02	6.61E-05	No	
cg19572487	5.59E-07	**1.24E-08**	**2.23E-08**	**1.92E-11**	Yes	2.20E-30
cg12513616	8.50E-07	1.11E-07	**1.75E-09**	**2.10E-09**	Yes	2.40E-19
cg24859433	1.86E-07	4.78E-06	**7.66E-10**	**3.15E-08**	Yes	8.40E-12
cg11207515	1.12E-05	1.58E-07	**6.60E-10**	**7.21E-11**	Yes	2.10E-09
cg11660018	6.65E-05	8.06E-07	**8.63E-08**	2.24E-06	Yes	4.30E-26
cg21611682	6.79E-07	9.48E-06	**1.11E-08**	2.69E-07	Yes	5.10E-13
cg23161492	2.90E-05	8.60E-06	**1.59E-08**	7.60E-06	Yes	9.50E-10
cg12835048	7.91E-05	2.13E-06	5.37E-05	**1.81E-09**	No	
cg00765233	3.72E-04	1.26E-05	1.51E-04	**9.89E-08**	No	
cg03055271	2.06E-01	1.89E-01	6.02E-02	**6.19E-08**	No	
cg21177558	5.10E-01	1.43E-01	1.21E-01	**6.27E-08**	No	
cg07826859	1.94E-06	1.39E-04	3.31E-05	**7.14E-08**	Yes	0.0057
cg10549831	2.47E-02	1.08E-04	2.19E-03	**5.14E-08**	No	
cg15159987	4.52E-04	5.83E-07	1.47E-03	**4.67E-09**	No	
cg02685484	4.35E-03	4.83E-04	9.70E-03	**6.19E-08**	No	
cg05552183	1.45E-02	6.06E-03	8.51E-02	**9.14E-08**	No	
cg12999636	2.26E-04	5.57E-07	6.54E-05	**6.35E-09**	No	
cg26754262	3.49E-03	3.91E-07	6.14E-05	**3.74E-11**	No	
cg21232704	5.87E-04	8.82E-06	6.04E-03	**1.80E-08**	No	
cg17372758	2.02E-05	6.80E-07	1.51E-06	**2.31E-09**	Yes	1.30E-05
cg18139806	6.52E-03	4.11E-06	1.01E-02	**4.05E-08**	No	
cg22132788	7.10E-04	2.21E-06	6.83E-05	**3.61E-08**	Yes	1.80E-07
cg06235438	2.54E-05	4.52E-07	5.30E-05	**1.51E-08**	Yes	6.60E-06
Total	18	36	29	46	60	
Total Replicated	17 (94.4%)	26 (72.2%)	27 (93.1%)	29 (63%)	37 (61.7%)	

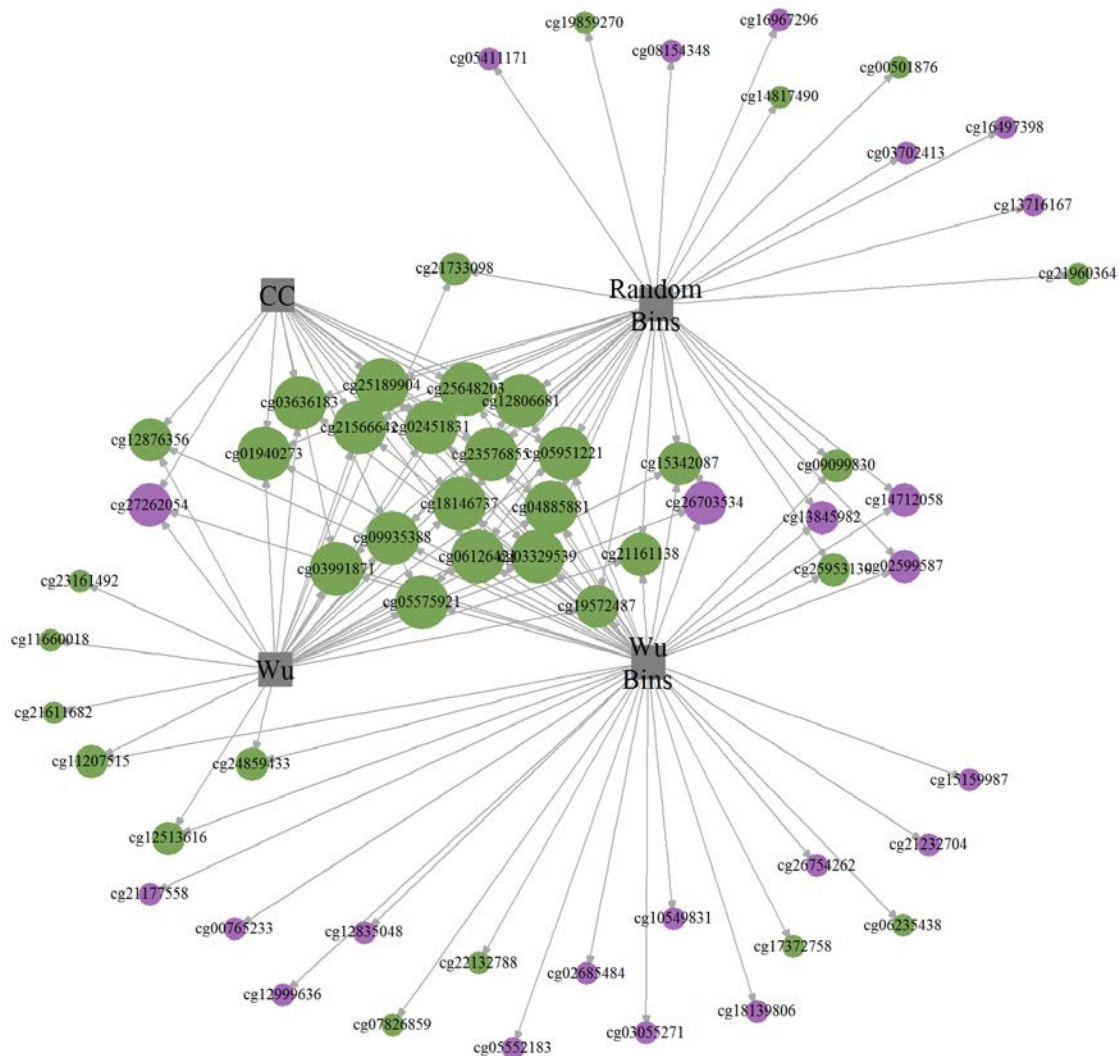

Web Figure 11. Illustrating the CpG sites identified as associated with smoking in the ARIES data, for the different methods. Methods are represented by gray squares, CpG sites are the circles. Arrows connect the methods with the CpG sites they identified. The size of the circle indicates how many methods identified that CpG site. Green circles indicate that the CpG site was replicated in one of the studies covered by Joehanes et al.⁶; purple indicates it was not replicated.

3.7 IPW Results

Web Table 20. The number of CpG sites associated with smoking (ex or current) identified in the regression analysis for the IPW models, for both missingness mechanisms (MM). We report the number of these which were also found in the analysis on the complete data set (presented with the percentage, i.e. the true positive rate) and the number of those which were not found in the analysis on the complete data set (presented with the percentage of those found which were “incorrect”, i.e. the false positive rate). The table shows the results of the 10 repeats on the full (unreduced) data set and is an extension of Table 1 in the main text.

	MM1				MM2			
	Associated CpGs			SE of Coefficients (Mean (SD))	Associated CpGs			SE of Coefficients (Mean (SD))
	Total	No. in Complete (% of Complete)	No. Not in Complete (% of Total Found)		Total	No. in Complete (% of Complete)	No. Not in Complete (% of Total Found)	
Complete Data	298	-	-	0.0953 (0.0081)	298	-	-	0.0953 (0.0081)
Naive IPW	144.5	44.2 (14.8%)	100.3 (67.7%)	0.1611 (0.0407)	288	136.9 (45.9%)	151.1 (49.9%)	0.1076 (0.0117)
Fixed IPW	124	59 (19.8%)	65 (49.9%)	0.1282 (0.0201)	246	115.2 (38.7%)	130.8 (50.4%)	0.1090 (0.0120)

References

1. Aryee MJ, Jaffe AE, Corrada-Bravo H, et al. Minfi: a flexible and comprehensive Bioconductor package for the analysis of Infinium DNA methylation microarrays. *Bioinformatics* 2014;**30**:1363-9.
2. Jaffe AE, Murakami P, Lee H, et al. Bump hunting to identify differentially methylated regions in epigenetic epidemiology studies. *International Journal of Epidemiology* 2012;**41**:200-9.
3. R Core Team. *R: A Language and Environment for Statistical Computing*. Vienna, Austria: R Foundation for Statistical Computing; 2016.
4. Peduzzi P, Concato J, Kemper E, Holford TR, Feinstein AR. A simulation study of the number of events per variable in logistic regression analysis. *Journal of Clinical Epidemiology* 1996;**49**:1373-9.
5. Hardt J, Herke M, Leonhart R. Auxiliary variables in multiple imputation in regression with missing X: a warning against including too many in small sample research. *BMC Medical Research Methodology* 2012;**12**:1.
6. Joehanes R, Just AC, Marioni RE, et al. Epigenetic signatures of cigarette smoking. *Circulation: Cardiovascular Genetics* 2016;**9**:436-47.
